# Eribulin for patients with metastatic breast cancer in England 2012-2021: Survival outcomes and 30-day mortality

**DOI:** 10.1016/j.breast.2026.104878

**Published:** 2026-07-19

**Authors:** Zhe Wang, Lorna Roden, Rebecca Watters, Bonnie E. Shook-Sa, Chris Twelves, David Dodwell

**Affiliations:** a Oxford Population Health, University of Oxford, UK; b University of Leeds, UK

## Abstract

**Background:**

Eribulin was approved for patients with metastatic breast cancer (MBC) following anthracycline and taxane therapy based on modest survival gains in trial populations. We considered survival outcomes and 30-day post-chemotherapy mortality among all NHS patients under Cancer Drugs Fund eligibility criteria, treated with eribulin in England between January 2012 and August 2021.

**Methods:**

Overall survival (OS) was estimated from start of eribulin using Poisson and Kaplan–Meier methods. Associations between patient characteristics and mortality were considered using Poisson and binomial regression. Thirty-day mortality was defined from the start of the most recent chemotherapy cycle.

**Results:**

6842 patients with MBC who initiated eribulin were identified. 88% of patients died during follow-up. Median OS was 8.9 months (95% CI 8.7–9.2); Survival was longer in patients with ER-positive disease (9.7 vs 7.4 months - compared to ER-negative) and strongly associated with performance status (PS0: 11.0; PS2+: 5.3 months). Adjusted mortality was lower in older patients, and substantially higher in those with poorer performance status (PS2+ vs PS0 RR 1.96, 95% CI 1.77–2.16). Overall, 17.2% patients died within 30 days of a cycle of chemotherapy, with higher risks in patients with poorer performance status, of younger age, and shorter interval since primary diagnosis.

**Conclusions:**

In routine NHS care in England, survival following eribulin was shorter and early mortality higher than reported in clinical trials, particularly among patients with impaired performance status. Careful patient selection and transparent discussion of risks are essential.

## Highlights

• The median survival for patient receiving eribulin was 8.9 months, substantially less than reported in the pivotal randomised trials.
• Younger patients (<50 years) had shorter survival than older patients.
• Performance status (PS) was strongly associated with outcome, with a two-fold increase in mortality among patients with PS ≥ 2 compared to PS 0.
• **T**he risk of 30-day mortality was 8.8%, much higher than those reported in clinical trials of eribulin.

## 1 Introduction

Eribulin (Halaven, eribulin mesylate) is a cytotoxic agent that targets microtubule growth. It is a synthetic analogue of the marine product Halichondrin B, derived initially from sea sponges.

Eribulin is used in people with metastatic breast cancer (MBC) and much less commonly in those with sarcoma. In MBC the pivotal randomised trials which supported the introduction of eribulin were the EMBRACE (305) study comparing eribulin to treatment of physician's choice in those with heavily pre-treated disease and the 301-study comparing eribulin to capecitabine in a less heavily pre-treated population. In a pooled analysis of these two trials including 1864 patients, there was a modest improvement in overall survival in patients receiving eribulin compared to standard of care. Treatment with eribulin was associated with apparent benefits in survival across all patient subgroups defined by ER/PR/HER2 status, degree of organ involvement, and prior taxane responsiveness [1].

Following European Medicines Agency (EMA) approval in March 2011 eribulin was licensed for use in metastatic breast cancer after anthracycline and taxane chemotherapy, and in the UK became available via the Cancer Drugs Fund (CDF) in 2011 [2]. NICE approval for use in locally advanced/metastatic breast cancer after ≥2 chemotherapy regimens was confirmed in December 2016 [3]. We report a large population-based observational study of 6842 patients with metastatic breast cancer treated with eribulin, during the period of eligibility criteria stipulated by the CDF. We considered overall survival outcomes and 30-day mortality following chemotherapy.

## 2 Methods

### 2.1 Study population

The National Cancer Registration and Analysis Service (NCRAS) and its preceding organisations register all patients diagnosed with cancer in England. These registrations are linked to other NCRAS-supported datasets to provide information on other variables including tumour site, year of diagnosis, age at diagnosis, ethnicity, staging, treatments and dates of emigration and death [4].

The Systemic Anti-Cancer Therapy dataset (SACT) constituted the originating data source for this analysis and provided detailed information on chemotherapy regimens, drugs, dose reductions, dose delays, treatment intent, performance status, height, weight, hospital trust, region and responsible consultant. After pilot work from 2010 to address feasibility, uploading of data to the SACT dataset started in April 2012 and became mandatory in the NHS in England from April 2014 [5].

Data were collated and checked for all patients registered in SACT with a breast cancer diagnosis and who started eribulin between 1 January 2012 and 31 August 2021. There were no restrictions according to age, sex or previous cancer diagnosis.

### 2.2 Method of analysis

Patients were included in the study from the start of eribulin until the earliest of date of death, emigration, or 31 August 2021.

Mortality rates were estimated by the ratio of the number of observed deaths to the number of person-years at risk. Overall survival from the start of eribulin was then estimated in intervals of one month. The extent to which the mortality rate varied with available patient and tumour characteristics was studied using Poisson regression. Confidence intervals and significance tests were calculated assuming that the numbers of observed deaths followed a Poisson distribution and the numbers of person years were fixed. In supplemental analyses, mortality risk at 6- and 12-months from the start of eribulin was compared by patient and tumour characteristics using Kaplan-Meier methods.

We also analysed the risk of 30-day mortality, considering the number of patients who died within 30 days of the start of the most recent cycle of chemotherapy compared with the number of patients starting that cycle. The variation in 30-day mortality risk by patient and tumour characteristics was studied using binomial regression. Confidence intervals and significance tests were calculated assuming that the numbers of observed deaths followed a binomial distribution and the numbers of patients were fixed. Calculations were carried out using Stata version 15.1 and R version 4.5.1.

### 2.3 Literature search

A literature search was conducted to identify clinical trials of any size, and observational studies including >250 patients, published from 1st January 2015 to 11th September 2025, which considered survival outcomes following the use of eribulin in metastatic breast cancer.

## 3 Results

### 3.1 Characteristics of the study population

**Fig. 1:**
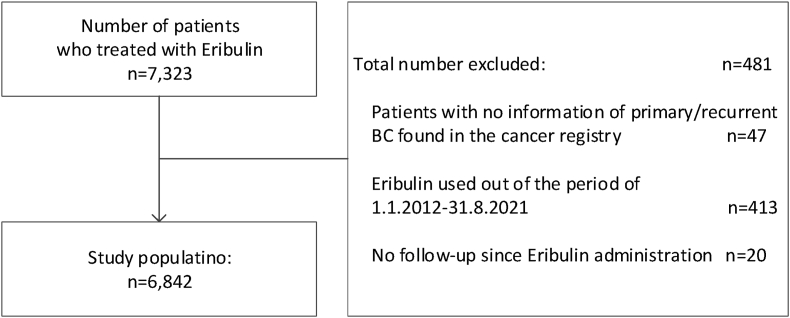
– Derivation of study population The most recent recorded date of death in the dataset is 31.8.2021.

A cohort of 7323 patients who had received eribulin were initially identified of whom 6842 were eligible for the study (Fig. 1). Patient, tumour and treatment characteristics are provided in Table 1 which includes data incompleteness. Of included patients 75.8% were aged ≥50 at start of treatment, 89.3% were of white ethnicity (Table S1), 0.6% were men, 87.8% had a performance status of 0 or 1, 59.5% were overweight or obese, 60.2% had ER positive tumours and 28.4% had HER-2 positive disease.

**Table 1:** Patient tumour and treatment characteristics.

Variable	Category	ER status (%)			*p*	Total
		Negative	Positive	Unknown		
Total	-	2096	4119	627		6842
Year of Eribulin	2012	289 (13.8)	386 (9.4)	156 (24.9)	<0.001	831 (12.1)
administration	2014	438 (20.9)	812 (19.7)	197 (31.4)		1447 (21.1)
	2016	461 (22.0)	1004 (24.4)	152 (24.2)		1617 (23.6)
	2018	516 (24.6)	1094 (26.6)	83 (13.2)		1693 (24.7)
	2020	392 (18.7)	823 (20.0)	39 (6.2)		1254 (18.3)
Age at Eribulin	<50	573 (27.3)	952 (23.1)	131 (20.9)	0.002	1656 (24.2)
	50-59	630 (30.1)	1376 (33.4)	212 (33.8)		2218 (32.4)
	60-69	550 (26.2)	1114 (27.0)	189 (30.1)		1853 (27.1)
	70+	343 (16.4)	677 (16.4)	95 (15.2)		1115 (16.3)
Sex	M	6 (0.3)	30 (0.7)	7 (1.1)	0.05	43 (0.6)
	F	2090 (99.7)	4089 (99.3)	620 (98.9)		6799 (99.4)
Race	White	1824 (87.0)	3719 (90.3)	565 (90.1)	<0.001	6108 (89.3)
	Black	100 (4.8)	114 (2.8)	23 (3.7)		237 (3.5)
	Other/unknown	172 (8.2)	286 (6.9)	39 (6.2)		497 (7.3)
Performance	0	463 (29.8)	963 (29.9)	131 (30.6)	0.99	1557 (29.9)
	1	903 (58.1)	1876 (58.2)	232 (54.2)		3011 (57.9)
	2+	187 (12.0)	383 (11.9)	65 (15.2)		635 (12.2)
	Unknown∗	543	897	199		1639
BMI (nearest)	Underweight <18.5	50 (2.7)	102 (2.7)	16 (3.0)	0.49	168 (2.7)
	Healthy <25.0	683 (36.2)	1432 (37.9)	229 (43.4)		2344 (37.9)
	Overweight <30.0	625 (33.2)	1256 (33.2)	148 (28.0)		2029 (32.8)
	Obesity 30+	527 (28.0)	989 (26.2)	135 (25.6)		1651 (26.7)
	Unknown	211	340	99		650
HER2 status	Negative	854 (69.3)	1675 (75.6)	40 (28.8)	<0.001	2569 (71.6)
	Positive	379 (30.7)	542 (24.4)	99 (71.2)		1020 (28.4)
	Unknown	863	1902	488		3253
Charlson	0	1505 (85.4)	2692 (88.1)	337 (89.6)	0.02	4534 (87.3)
comorbidity	1	108 (6.1)	158 (5.2)	16 (4.3)		282 (5.4)
index	2+	150 (8.5)	204 (6.7)	23 (6.1)		377 (7.3)
	Unknown	333	1065	251		1649
Year since	0-1	421 (20.1)	265 (6.4)	40 (6.4)	<0.001	726 (10.6)
primary BC	2-4	835 (39.8)	1185 (28.8)	111 (17.7)		2131 (31.1)
diagnosis	5-9	490 (23.4)	1386 (33.6)	238 (38.0)		2114 (30.9)
	10+	350 (16.7)	1283 (31.1)	238 (38.0)		1871 (27.3)
Vital status	Alive	250 (11.9)	535 (13.0)	33 (5.3)	0.29	818 (12.0)
at 31-08-2021	Dead	1846 (88.1)	3582 (87.0)	594 (94.7)		6022 (88.0)
	Loss of follow-up	-	2	-		2

Unknown categories were excluded from the calculation of column percentages and from the tests for homogeneity.

There were records of chemotherapy prior to eribulin in 94.2% of patients and 48.4% of patients received further chemotherapy after eribulin (Table S2). The majority (88%) of patients without a previous recording of chemotherapy within the SACT dataset, prior to eribulin were treated in the early calendar year period of the cohort when SACT records were incomplete (Table S3). The majority of patients received anthracycline and taxane based chemotherapy prior to the start of eribulin (Table S2) consistent with CDF eligibility criteria [2].

### 3.2 Overall survival

**Fig. 2:**
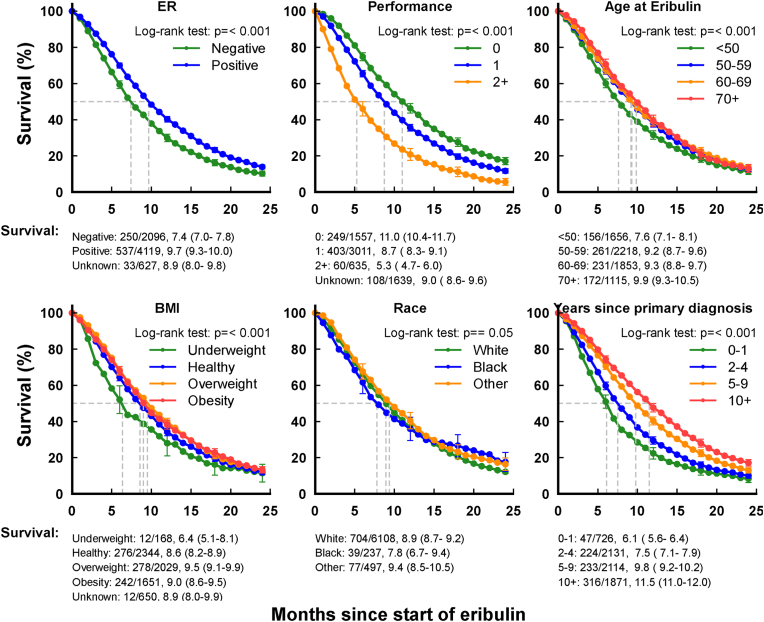
Survival from start of eribulin Performance status and body mass index (BMI) measured within 30 days of the start of eribulin administration. Medians and (interquartile ranges) provided.

Overall, 88.0% of patients died during the study period. The median survival from start of eribulin was 8.9 months (95% Confidence Interval [CI] 8.7-9.2 months). Survival was significantly shorter for patients with ER negative compared to ER positive disease (7.4 [7.0-7.8] vs 9.7 [9.3-10] months), for patients <50 years of age compared to older patients (<50 years – 7.6 [7.1-8.1], 50-59 years – 9.2 [8.7-9.6], 60-69 years – 9.3 [8.8-9.7], 70+ years – 9.9 [9.3-10.5] months), for those with a poorer performance status (PS), PS0 – 11.0 [10.4-11.7], PS1 – 8.7[8.3-9.1], PS2+ - 5.3[4.7-6.0] months) and those classed as underweight – 6.4[5.1-8.1], healthy weight – 8.6[8.2-8.9] months. A shorter time period between first diagnosis of breast cancer and start of eribulin was also strongly associated with shorter survival (0-1 year – 6.1 [5.6-6.4], 10+ years – 11.5[11.0-12.0] months (Fig. 2).

**Fig. 3:**
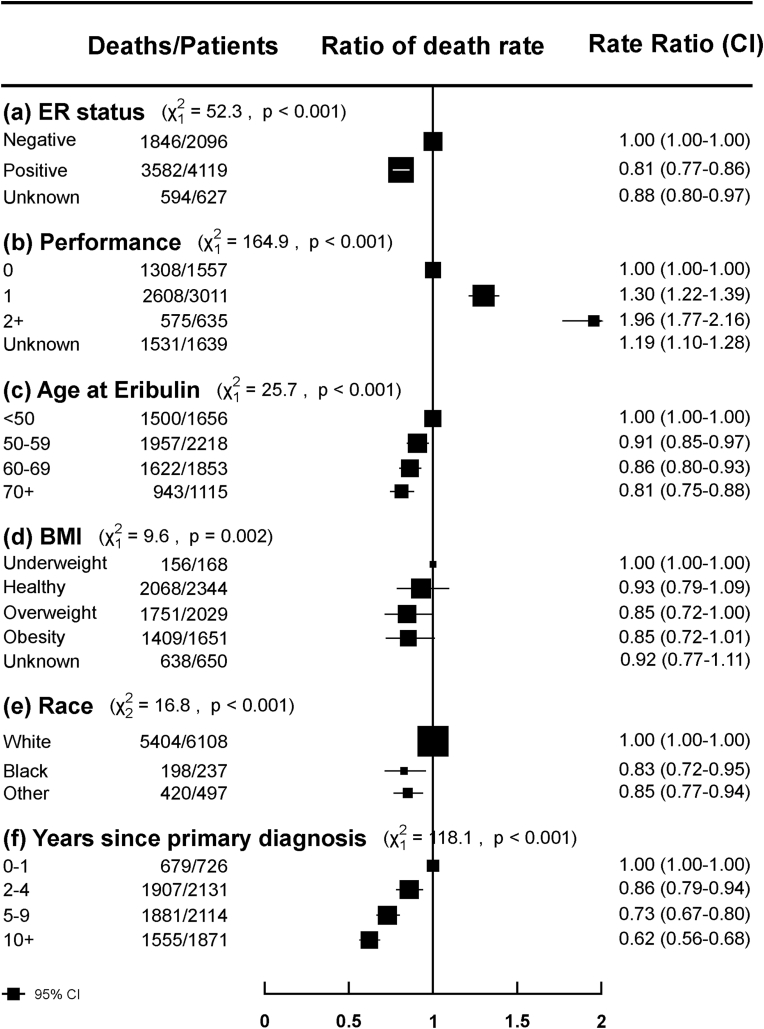
Adjusted mortality rates following start of eribulin. Adjusted rate ratios presented were estimated after adjustment for age, race, ER status, performance status, and BMI, and trend tests were conducted for all variables except race. In all analyses, patients with unknown status were excluded.

Fig. 3 provides adjusted mortality rate ratios (RR) to demonstrate the independent associations of mortality with available variables. ER positive status (RR = 0.81 [0.77-0.86], and older age (50-59 years, RR = 0.91 [0.85-0.97], 60-69 years, RR = 0.86 [0.80-0.93], 70+ years, RR = 0.81 [0.75-0.88]) were associated with lower mortality. Higher mortality was associated with poorer performance status (PS1, RR = 1.3 [1.22-1.39], PS2+, RR = 1.96 [1.77-2.16] compared with PS0).

Supplemental Fig. 1 presents cumulative risks of mortality and risk ratios at 6- and 12-months from the start of eribulin by patient and tumour characteristics. Of the variables examined, performance status had the strongest association with mortality at both 6 and 12 months. Cumulative risk of mortality at 6-months was 25.2% for PS0, 34.6% for PS1, and 54.1% for PS2+, for estimated risk ratios of 1.37 [1.24-1.52] (PS1 vs PS0) and 2.14 [1.91-2.40] (PS2+ vs PS0). The magnitude of this association attenuated with time, with estimated 12-month mortality risks of 53.8%, 64.4%, and 79.3% for PS0, PS1, and PS2+, respectively (PS1 risk ratio = 1.20 [1.13-1.27], PS2+ risk ratio = 1.47 [1.38-1.57] compared with PS0).

### 3.3 30-day mortality risk

There were 604 deaths within 30 days of the start of the most recent cycle of eribulin (8.8%). 30-day mortality risk following eribulin was strongly associated with poorer performance status (PS0 = 5.1%, PS1 = 9.1%, PS2+ = 17.8%, P < 0.001), younger age at start of eribulin (<50=11.2%, >70 = 6.3%, P < 0.001) and a shorter period between initial breast cancer diagnosis and treatment (0-1 year = 12.1%, 10+ years = 6.4%) (Table 2). Multivariate analysis confirmed the associations between ER negative status, poorer performance status, younger age at treatment and shorter time between primary breast cancer diagnosis and eribulin treatment with higher 30-day mortality after eribulin (Supplemental Fig. 2) Table S4 provides 30-day mortality values for drugs given after eribulin.

**Tables 2:** 30-day mortality after eribulin chemotherapy.

	Category	30-day mortality (%)	*p*
Total	-	604/6842 (8.8)	
ER status	Negative	226/2096 (10.78)	<0.001
	Positive	315/4119 (7.65)	
	Unknown	63/627 (10.05)	
Performance	0	79/1557 (5.07)	<0.001
	1	275/3011 (9.13)	
	2+	113/635 (17.80)	
	Unknown	137/1639 (8.36)	
Age at Eribulin	<50	185/1656 (11.17)	<0.001
	50-59	208/2218 (9.38)	
	60-69	141/1853 (7.61)	
	70+	70/1115 (6.28)	
BMI (nearest)	Underweight <18.5	24/168 (14.29)	0.03
	Healthy <25.0	221/2344 (9.43)	
	Overweight <30.0	162/2029 (7.98)	
	Obesity 30+	148/1651 (8.96)	
	Unknown	49/650 (7.54)	
Ethnicity	White	550/6108 (9.00)	0.06
	Black	24/237 (10.13)	
	Other	30/497 (6.04)	
Years since primary BC	0-1	88/726 (12.12)	<0.001
diagnosis	2-4	226/2131 (10.61)	
	5-9	171/2114 (8.09)	
	10+	119/1871 (6.36)	

### 3.4 Dose reductions and early treatment discontinuation

Eribulin was discontinued early in 33.7% (1362) patients, 35.4% of patients experienced a dose reduction and 38.5% experienced one or more delays during the course of treatment (Table S5)

### 3.5 Treatments after eribulin

Following discontinuation of eribulin, patients whose date of eribulin initiation was before 2020, received the following drugs as single agents in subsequent lines of therapy – vinorelbine (23.1%), paclitaxel (14.4%), carboplatin (15.4%), capecitabine (8.4%) and epirubicin (10.4%). Other combination regimens were also received (Table S2).

## 4 Discussion

### 4.1 Principal findings

This population-based observational study of 6842 patients with metastatic breast cancer treated with eribulin is the largest to our knowledge and has the advantages of inclusion of all patients treated in the English NHS over a 9-year time period with eligibility criteria mandated by national Cancer Drug Fund (CDF) requirements. These criteria included the stipulation that all patients had received two previous chemotherapy regimens for advanced disease and had received previous taxane and anthracycline-based chemotherapy for either early or advanced disease [2,3].

There was an overall median survival of 8.9 months. Patients with ER-positive disease demonstrated significantly longer survival compared with those with ER-negative cancers, as did those with a longer interval between first diagnosis of breast cancer and treatment with eribulin. These observations are likely to relate to innate disease biology. Younger patients (<50 years) had shorter survival than older patients, an observation potentially related to a tendency for younger patients, with a greater burden of disease and symptoms to be treated with chemotherapy, compared to their older counterparts who may have been more likely to have received symptomatic and supportive care only but this interpretation is inferential. Performance status (PS) was most strongly associated with outcome, with a two-fold increase in mortality among patients with PS ≥ 2 compared to those with a better PS, underscoring the need to consider avoidance of overtreatment in patients with declining performance status.

**T**he risk of 30-day mortality was 8.8%, higher than those reported in clinical trials of eribulin, where patients with a poor performance status, significant comorbidities or of older age are likely to have been excluded.

The use of chemotherapy before and after eribulin was consistent with national (CDF) guidance and most patients received anthracycline- and taxane-based regimens before eribulin, and 48.4% received further chemotherapy after discontinuation.

Over a third of patients required dose reductions or delays reflecting the toxicity profile of eribulin, but most modifications did not preclude treatment continuation.

### 4.2 Strengths and weaknesses

Major strengths of this study include its large size, inclusion of all English NHS patients over a 9-year period, treatment according to CDF eligibility criteria and availability of important patient, tumour and treatment characteristics including performance status and BMI. The study also included men.

Weaknesses include a lack of information on tumour response, frailty, quality of life (QL), and non-fatal treatment toxicity. Cancer registry data were incomplete for some variables and source data verification is not possible when using large scale cancer registry data. The SACT dataset has limitations from 2012 to 2016, explaining some gaps in the recording of prior chemotherapy.

We cannot conclude whether, and to what extent, eribulin was beneficial as causality cannot be easily addressed when analysing observational data, and tumour response, response duration and QL data were not available. The associations we identified should be considered in the context of the totality of evidence from this, other observational studies, and prospective trials (Tables S6 and S7).

### 4.3 Comparison with existing literature

The median OS of 8.9 months, observed in this study was substantially shorter than that reported from the pivotal pooled EMBRACE and Study 301 analyses (15.2 months) [1] and other prospective trials (Table S6), which have reported median OS values in the range of 8-28 months. This range is likely to encourage underestimation of the survival pattern, as in 8 of the 56 trials identified, the median OS had not been reached at the time of reporting although median follow up durations varied from 24 to 45 months.

Observational cohorts have reported median OS values of between 7 and 21 months, depending on previous therapy, PS, and receptor subtype (Table S7).

Toxicity was recorded in many different ways in published studies, limiting cross-study comparisons. In respect of possible toxic deaths, the EMBRACE trial reported that 4% of patients experienced a fatal adverse event but in only 1% of cases was this felt to be caused by eribulin [6]. In the 301-study fatal adverse events, within 30 days of the last dose, occurred in 4.8% of eribulin-treated patients [7]. This is in contrast to the 8.8% we identified. This may reflect our population being less fit and being treated in later line settings than in these pivotal trials.

### 4.4 Implications

This study provides the most comprehensive assessment to date of survival outcomes following eribulin in routine clinical practice. The findings highlight the strong association between poor performance status and lower survival, as well as the poorer survival, and higher 30-day mortality risk, than were reported in the prospective EMBRACE and Study 301 trials [6,7]. This reflects the need to inform patients that likely outcomes following eribulin treatment are poorer, and risks of mortality associated with treatment are higher, than the outcomes determined within prospective studies.

Randomized evidence provides information on relative and absolute treatment effects in trial-selected patients but whether, and to what extent, these effects apply in routine care is unknowable although often assumed. It is recognised that patients commonly overestimate both their own survival expectation and the benefits of palliative systemic therapies [8]. A study that compared survival patterns identified within randomised trials with those seen in parallel real-world datasets, also found that the disparity between survival outcomes increased where survival estimates were lowest [9].

### 4.5 Unanswered questions and future research

The prediction of benefit and toxicity from eribulin at patient level is substantially error prone. The high early mortality and poor survival we observed underline a need for improved techniques to identify patients for whom chemotherapy is futile or harmful, but this is profoundly difficult as comparative analyses with best supportive care are unfeasible. There are few large-scale, real-world data studies such as ours, and it is likely that disparities between clinical trial and real-world outcomes apply to other interventions in people with advanced or metastatic disease, not limited to breast cancer.

Our results do not challenge the “efficacy” of eribulin. Rather, they highlight that use of new treatments such as eribulin in routine patient populations, quite different to those included in clinical trials, is likely to be associated with poorer outcomes. The challenge for clinicians is to widen the use of new agents beyond candidates for clinical trials in a manner that is safe and effective.

The routine capture of validated measures of tumour response, consistent definitions of ‘line of therapy’, quality of life, frailty and patient-prioritised outcomes in observational studies as well as clinical trials is needed to better distinguish benefit from harm.

How trial evidence is incorporated into practice, particularly in frailer populations excluded from randomised studies, requires robust ongoing assessment.

Without these further efforts, systemic therapy given near end-of-life, risks becoming default practice rather than an informed choice.

## Contributors

DD, developed the study concept and design. ZW, BES, DD contributed to the data collection and collation. ZW, BES, performed the data analysis. LR, RW, DD did the literature review. All authors participated in data interpretation and manuscript preparation. All authors read and approved the final version. DD is the guarantor and attests that all listed authors meet authorship criteria, and that no others meeting the criteria have been omitted.

Approval for the study was granted by Public Health England's Office for Data Release (reference ODR 1920_080) and ethical approval was obtained (REC 19/NS/0057). Informed consent from individual participants was not required.

## Funding

Funding was provided by 10.13039/501100000289Cancer Research UK (grants C7852–A25447 and the 10.13039/501100000769University of Oxford. These funding bodies had no role in study design; in the collection, analysis, and interpretation of data; in the writing of the report; or in the decision to submit the article for publication.

Transparency: The corresponding author (the manuscript's guarantor) affirms that this manuscript is an honest, accurate, and transparent account of the study being reported; that no important aspects of the study have been omitted; and that any discrepancies from the study as planned (and, if relevant, registered) have been explained.

Dissemination to participants and related patient and public communities: The results have already been presented to representatives of Independent Cancer Patients’ Voice. After publication, they will be freely available both to stakeholders and to the broader public.

## CRediT authorship contribution statement

**Zhe Wang:** Conceptualization, Data curation, Formal analysis, Methodology, Project administration, Writing – original draft, Writing – review & editing. **Lorna Roden:** Project administration, Writing – original draft, Writing – review & editing. **Rebecca Watters:** Investigation, Writing – original draft, Writing – review & editing. **Bonnie E. Shook-Sa:** Formal analysis, Investigation, Methodology, Writing – original draft, Writing – review & editing. **Chris Twelves:** Conceptualization, Investigation, Writing – original draft, Writing – review & editing. **David Dodwell:** Conceptualization, Data curation, Funding acquisition, Investigation, Project administration, Supervision, Validation, Writing – original draft, Writing – review & editing.

## Competing interest statement

Competing interests: All authors have completed the ICMJE uniform disclosure form at www.icmje.org/disclosure-of-interest and declare: support from Cancer Research UK and the University of Oxford for the submitted work; there are no financial relationships with any organisations that might have an interest in the submitted work in the previous three years and no other relationships or activities that could appear to have influenced the submitted work.
