# Supplementary material for: Eribulin for patients with metastatic breast cancer in England 2012-2021: Survival outcomes and 30-day mortality
Source: Breast. 2026 Jul 19;89:104878. doi: 10.1016/j.breast.2026.104878 (PMC13396751; doi:10.1016/j.breast.2026.104878)
Supplement: Multimedia component 1 [file mmc1.pdf]

SUPPLEMENTARY APPENDIX

CONTENTS

	**Page**
Table S1 Ethnicity distribution	2
Table S2 Drugs used before or after Eribulin	2
Table S3 Distribution of patients who had Eribulin as their first record	3
Figure S1 - Cumulative Risk of Death	4
Table S4 - Drugs used in patients who died within 30 days of their last cycle	5
Table S5 - Eribulin dose reductions and delays	6
Figure S2 -Multivariate analysis of 30-day mortality risk after eribulin	6
Table S6 - Meta-analyses, randomised controlled trials and early phase trials of eribulin	7
Table S7 - Observational, cohort and real-world studies of eribulin	20

Table S1 Ethnicity distribution

ETHNICITY	Freq.	Percent
WHITE BRITISH	5,733	83.79
ANY OTHER WHITE BACKGROUND	307	4.49
ANY OTHER ETHNIC GROUP	136	1.99
BLACK CARIBBEAN	97	1.42
BLACK AFRICAN	89	1.30
ASIAN INDIAN	87	1.27
ANY OTHER ASIAN BACKGROUND	70	1.02
ASIAN PAKISTANI	68	0.99
WHITE IRISH	59	0.86
ANY OTHER BLACK BACKGROUND	51	0.75
NOT KNOWN	44	0.64
CHINESE	29	0.42
ANY OTHER MIXED BACKGROUND	16	0.23
ASIAN BANGLADESHI	15	0.22
MIXED WHITE AND BLACK CARIBBEAN	15	0.22
MIXED WHITE AND ASIAN	9	0.13
WHITE	9	0.13
MIXED WHITE AND BLACK AFRICAN	8	0.12
**Total**	**6,842**	**100.00**

Table S2 Drugs used before or after Eribulin

6,449 patients who had chemotherapy records before administrating Eribulin	3,311 patients who had chemotherapy records after administrating Eribulin
**Five most commonly used drugs as single agents**	
Capecitabine (35.5%)	Vinorelbine (23.1%)
Paclitaxel (24.9%)	Carboplatin (15.4%)
Vinorelbine (9.4%)	Paclitaxel (14.4%)
Carboplatin (4.7%)	Epirubicin (10.4%)
Docetaxel (4.2%)	Capecitabine (8.4%)
**Taxane*, Anthracycline and Capecitabine regimens**	
Taxane contained (78.3%)	Taxane contained (24.8%)
Anthracycline contained (31.8%)	Anthracycline contained (23.5%)
Capecitabine contained (73.5%)	Capecitabine contained (12.2%)

*Taxane contained: any regimen includes PACLITAXEL or DOCETAXEL.

Anthracycline contained: any regimen includes EPIRUBICIN, DOXORUBICIN, MITOXANTRONE

Capecitabine contained: any regimen including CAPECITABINE (see below)

BEVACIZUMAB + CAPECITABINE
CAPECITABINE + CARBOPLATIN
CAPECITABINE + CISPLATIN + TRASTUZUMAB
CAPECITABINE + DOCETAXEL
CAPECITABINE + EPIRUBICIN
CAPECITABINE + EPIRUBICIN + OXALIPLATIN
CAPECITABINE + GEMCITABINE
CAPECITABINE + IRINOTECAN
CAPECITABINE + LAPATINIB
CAPECITABINE + NERATINIB
CAPECITABINE + OXALIPLATIN
CAPECITABINE + PACLITAXEL
CAPECITABINE + TRASTUZUMAB
CAPECITABINE + VINORELBINE
CISPLATIN + CAPECITABINE + EPIRUBICIN

Table S3 Distribution of patients who had Eribulin as their first record*

Year of Eribulin admin.	number	Percentage %
2012	142	36.13
2013	133	33.84
2014	47	11.96
2015	24	6.11
2016	17	4.33
2017	14	3.56
2018	6	1.53
2019	4	1.02
2020	4	1.02
2021	2	0.51
Total	393	100.00

*According to the NICE advanced BC guideline, patients who can be prescribed Eribulin must have undergone two preceding chemotherapies. However, there are 393 patients whose first chemotherapy was recorded as Eribulin because data collection of SACT started officially in April 2012. Therefore, when we analysed the pre-Eribulin regimens, the records from the first two years were excluded from the analysis. Similarly, the records from the last two years were excluded to investigate the chemotherapy regime after Eribulin, for the same reason.

Supplementary Figure 1 - Cumulative Risk of Death

Cumulative risk of death, 6 months

Characteristic		Risk	RR (95% CI)
ER status	Negative	40.6%	Reference
	Positive	30.1%	0.74 (0.69, 0.79)
	Unknown	36.3%	0.89 (0.79, 1.00)
Performance	0	25.2%	Reference
	1	34.6%	1.37 (1.24, 1.52)
	2+	54.1%	2.14 (1.91, 2.40)
	Unknown	33.0%	1.31 (1.17, 1.46)
Age at Eribulin	< 50	40.0%	Reference
	50-59	32.9%	0.82 (0.76, 0.90)
	60-69	32.4%	0.81 (0.74, 0.89)
	70+	29.4%	0.73 (0.66, 0.82)
BMI	Underweight	48.0%	Reference
	Healthy	35.6%	0.74 (0.63, 0.88)
	Overweight	31.7%	0.66 (0.56, 0.79)
	Obesity	32.1%	0.67 (0.56, 0.80)
	Unknown	35.1%	0.73 (0.61, 0.89)
Race	White	33.8%	Reference
	Black	38.5%	1.14 (0.96, 1.35)
	Other/unknown	32.5%	0.96 (0.84, 1.10)
Years since primary diagnosis	0-1	49.1%	Reference
	2-4	40.3%	0.82 (0.75, 0.90)
	5-9	29.3%	0.60 (0.54, 0.66)
	10+	25.8%	0.52 (0.47, 0.58)

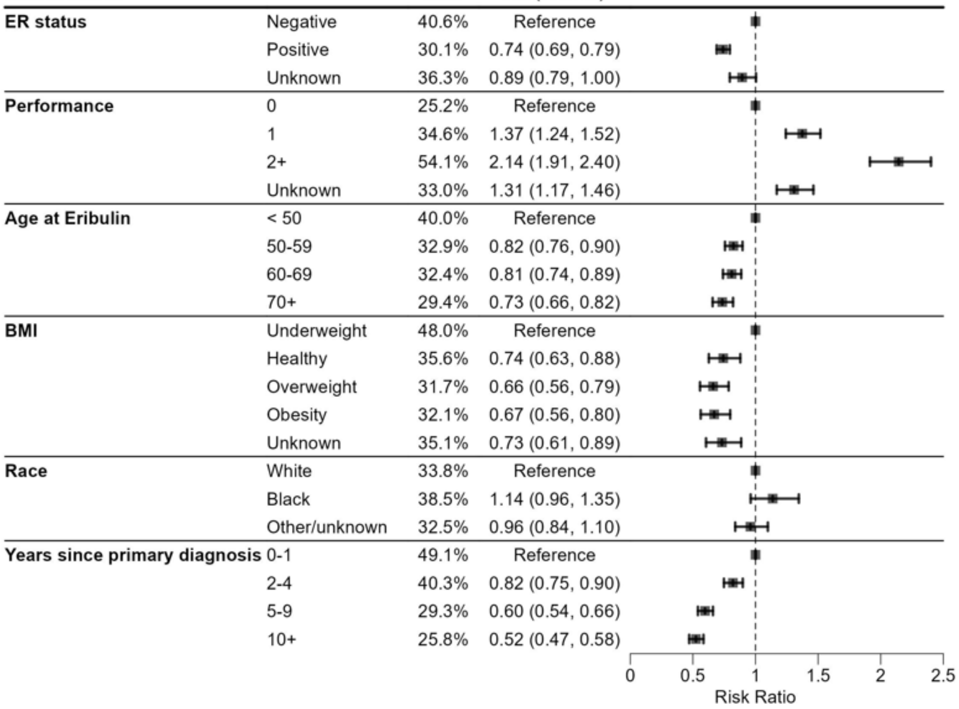

Cumulative risk of death, 12 months

Characteristic		Risk	RR (95% CI)
ER status	Negative	70.2%	Reference
	Positive	59.8%	0.85 (0.82, 0.89)
	Unknown	61.8%	0.88 (0.82, 0.94)
Performance	0	53.8%	Reference
	1	64.4%	1.20 (1.13, 1.27)
	2+	79.3%	1.47 (1.38, 1.57)
	Unknown	63.2%	1.17 (1.11, 1.25)
Age at Eribulin	< 50	68.5%	Reference
	50-59	62.0%	0.91 (0.86, 0.95)
	60-69	61.2%	0.89 (0.85, 0.94)
	70+	60.6%	0.89 (0.83, 0.94)
BMI	Underweight	72.2%	Reference
	Healthy	65.9%	0.91 (0.83, 1.01)
	Overweight	60.6%	0.84 (0.76, 0.93)
	Obesity	61.8%	0.86 (0.77, 0.95)
	Unknown	62.0%	0.86 (0.77, 0.96)
Race	White	63.3%	Reference
	Black	64.3%	1.01 (0.92, 1.12)
	Other/unknown	59.7%	0.94 (0.87, 1.02)
Years since primary diagnosis	0-1	77.7%	Reference
	2-4	70.5%	0.91 (0.86, 0.95)
	5-9	59.8%	0.77 (0.73, 0.81)
	10+	52.5%	0.68 (0.64, 0.72)

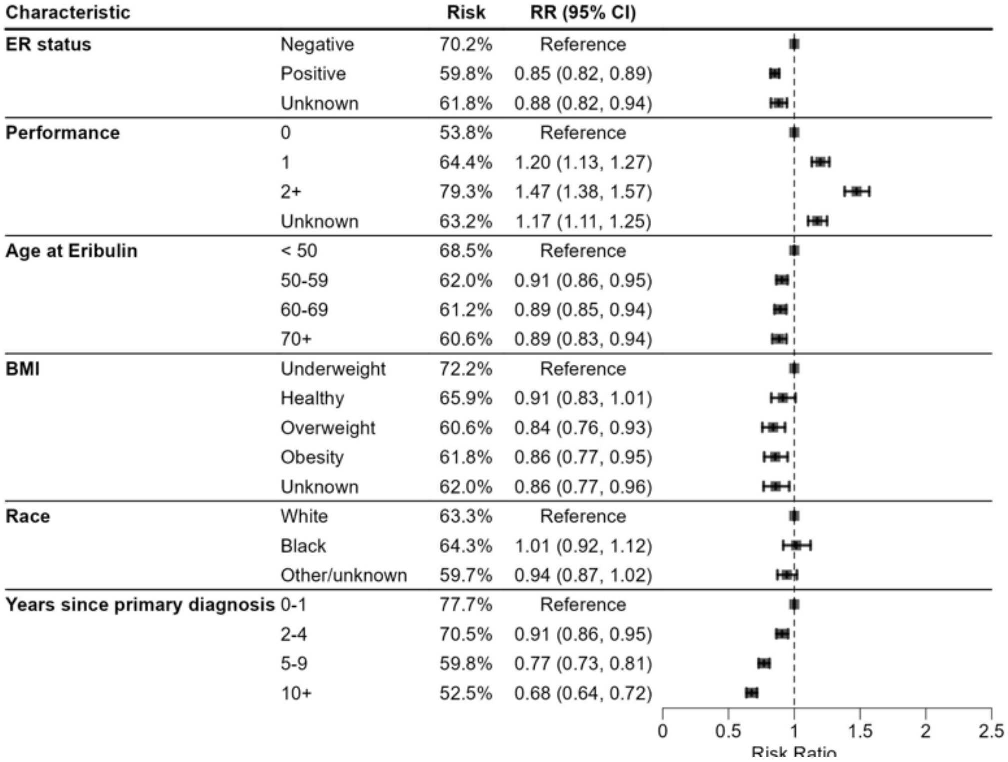

Table S4 - Drugs used in patients who died within 30 days of their last cycle

Drug used in last cycle	No of patients who died within 30 days of last cycle	Percentage (%)
Eribulin	604	51.2
Vinorelbine	132	11.2
Paclitaxel	91	7.7
Carboplatin	70	5.9
Epirubicin	68	5.8
Capecitabine	47	4.0
Other	167	14.2
Total	1179	100.0

Supplementary Figure 2 – Multivariate analysis of 30-day mortality risk after eribulin

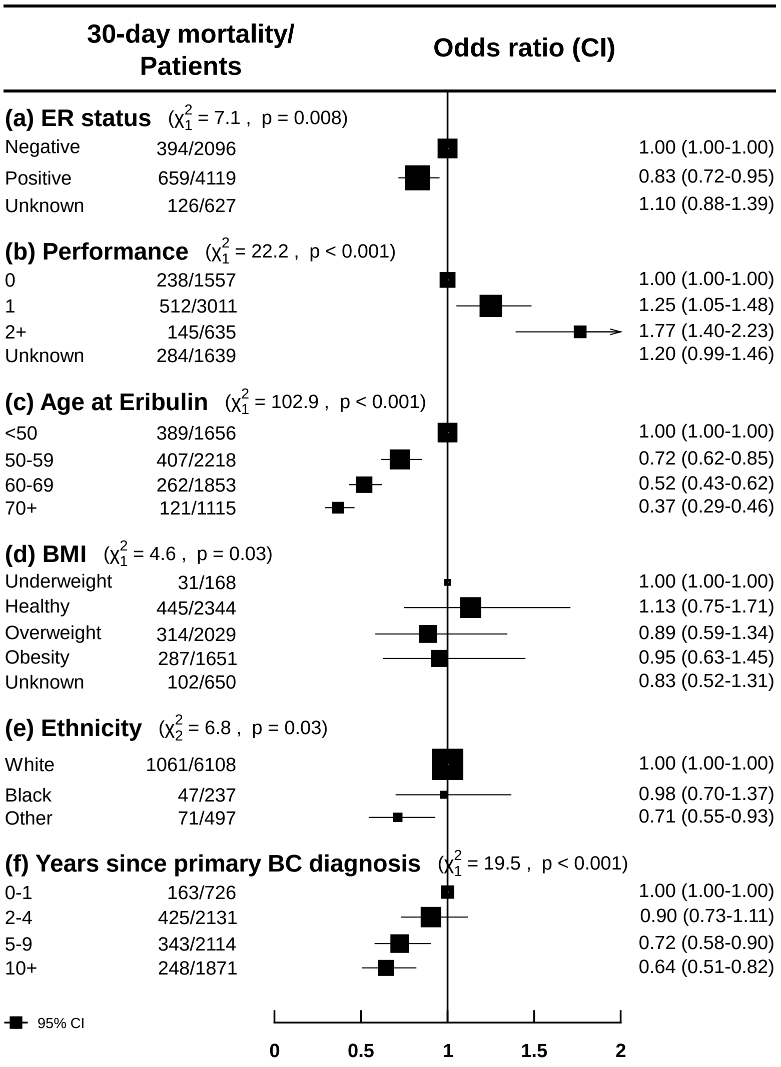

Table S5 - Eribulin dose reductions and delays

	Yes	No	Unknown
Eribulin stopped early	1362 (33.7)	2685 (66.3)	2795
Dose reduction	1843 (35.4)	3360 (64.6)	1639
Time delay	1278 (38.5)	2039 (61.5)	3525

Table S6 - Meta-analyses, randomised controlled trials and early phase trials of eribulin (56)

A search was performed on 11th September 2025 for studies published between 1st January 2015 until 11th September 2025 on patients with metastatic breast cancer where eribulin was compared to another drug in a meta-analyses or controlled trial. Studies were included if they were written in English, conducted in humans and included over 250 participants regardless of treatment arm. Studies without published survival data (overall survival or progression free survival) were excluded as were trials that repeated outcomes reported elsewhere and included here.

Meta-analyses, randomised controlled trials and early phase trials of eribulin (56)			
**Publication** (Newest first)	**Median age (years)**	**Summary of findings**	**Reported survival outcomes** for eribulin-treated patients (unless otherwise stated)
Yamashita T, Saji S, Takano T, et al. Trastuzumab-Pertuzumab Plus Eribulin or Taxane as First-Line Chemotherapy for Human Epidermal Growth Factor 2-Positive Locally Advanced/Metastatic Breast Cancer: The Randomized Noninferiority Phase III EMERALD Trial. *J Clin Oncol*. 2025;43(11):1302-1313.	56	This phase III randomised controlled trial of 446 patients with locally advanced/metastatic HER2-positive breast cancer compared eribulin and trastuzumab/pertuzumab (HP) with taxane and HP. The median follow up time was 35.7 months (0.3-66.5) with a median duration of eribulin of 28.1 weeks. The median PFS was 14.0 and 12.9 months in the eribulin and taxane group respectively (HR 0.95 (95% CI 0.76-1.19), confirming non-inferiority of eribulin. There were 60/224 deaths (26.8%), with 57/60 (95%) of deaths due to primary disease.	Median OS not reached.<br>PFS not reported.

Meta-analyses, randomised controlled trials and early phase trials of eribulin (56)			
**Publication** (Newest first)	**Median age (years)**	**Summary of findings**	**Reported survival outcomes** for eribulin-treated patients (unless otherwise stated)
Anampa JD, Flynn DL, Leary C, et al. Phase Ib Clinical and Pharmacodynamic Study of the TIE2 Kinase Inhibitor Rebastinib with Paclitaxel or Eribulin in HER2-Negative Metastatic Breast Cancer. *Clin Cancer Res*. 2025;31(2):266-277	60	This phase IB trial included 15 patients with HER2-negative metastatic breast cancer. They received eribulin in combination with rebastinib with a median follow up 15 months (3-39).	OS not reported. Median PFS 2.6 months (eribulin alone). Median PFS 2.8 months (eribulin plus rebastinib). (95% CI's not published).
Allsopp RC, Guo Q, Page K, et al. Circulating tumour DNA dynamics during alternating chemotherapy and hormonal therapy in metastatic breast cancer: the ALERT study. *Breast Cancer Res Treat*. 2024;206(2):377-385.	50	This study investigated circulating tumour DNA dynamics in 8 patients with advanced breast cancer. All patients received eribulin. 10% of patients had grade 3 adverse events. Dynamic changes in ctDNA were observed in short timescales between chemotherapy treatment and support the clinical benefit seen in individual patients.	OS not reported. Median PFS at 6 months 202 days (95% CI 135-undefined). Median PFS at 9 months 235 days (95% CI 235-undefined).
Gumusay O, Huppert LA, Magbanua MJM, et al. A phase Ib/II study of eribulin in combination with cyclophosphamide in patients with advanced breast cancer. Breast Cancer Res Treat. 2024 Jan;203(2):197-204.	56	44 women with metastatic cancer or unresectable breast cancer (of any receptor status) were enrolled in this expanded dose escalation study. Eribulin was given in combination with 600 mg/m^2 cyclophosphamide. The main outcome measure was to determine the maximum tolerated dose and to estimate the clinical benefit rate. The median duration of treatment was 14.7 weeks (1.8-53.3). 79.5% (35/44) had a partial response. No dose limiting toxicities were identified. 63.6% of patients experienced a grade 3 or 4 adverse event.	OS not reported. Median PFS 16.4 weeks (95% CI 13.8-21.1).
Landry CA, Blanter J, Ru M, et al. Results of a Phase Ib Study Investigating Durvalumab in Combination with Eribulin in Patients with HER2-Negative Metastatic Breast Cancer and Recurrent. Ovarian Cancer. Oncology. 2024;102(1):9-16.	63	[Abstract only] 9 patients with HER2-negative breast cancer or recurrent ovarian cancer at a single US institution were treated in a modified 3+3 design trial with escalating doses of eribulin and durvalumab until dose limiting toxicity, adverse events or disease progression. The primary outcome measure was the rate of dose limiting toxicity. The recommended dose of eribulin was 1.4 mg/m^2. The ORR was 55% (all partial response).	Median OS not reached. Median PFS 6.2 months. (95% CI's not published).
Kim SH, Im SA, Suh KJ, et al. Clinical activity of nivolumab in combination with eribulin in HER2-negative metastatic breast cancer: A phase IB/II study (KCSG BR18-16). Eur J Cancer. 2023 Dec;195:113386.	51	A phase II trial in 10 Korean hospitals of 99 women with metastatic HER2-negative breast cancer of eribulin and nivolumab (360 mg on day 1). The primary endpoint was 6-month PFS. The ORR was 41.1% (95% CI 30.8-52.0). 65.5% of patients experienced a grade 3 or 4 adverse event.	Median OS 17.9 months (95% CI 15.1-undefined; in HER2-negative ER-positive patients). Median OS 15.7 months (95% CI 11.0-21.9; in TNBC patients). (p=0.26)

Meta-analyses, randomised controlled trials and early phase trials of eribulin (56)			
**Publication**<br>(Newest first)	**Median age (years)**	**Summary of findings**	**Reported survival outcomes** for eribulin-treated patients (unless otherwise stated)
Kobayashi K, Masuda N, Mizuno T, et al. Phase II trial of biweekly administration with eribulin after three cycles of induction therapy in hormone receptor-positive, HER2-negative metastatic breast cancer (JACCRO BC-03). Breast Cancer Res Treat. 2023 Oct;201(3):409-415.	57	60 patients with ER-positive HER2-negative breast cancer in a number of Japanese hospitals received bi-weekly eribulin after a three-cycle induction treatment (of 1.4 mg/m^2 eribulin on days 1 and 8 of a 21-day cycle for 3 cycles). The primary outcome measure was PFS in patients who continued therapy after induction. Median PFS was 15.21 weeks (95% CI 9.71-22.14) and the response rate was 17.5%. Grade 3 or higher neutropenia was experienced by 50% of the patients.	Median OS 21.39 months (95% CI 18.89-32.89; in patients continuing to bi-weekly treatment after induction).
Nelson BE, Saleem S, Damodaran S, et al. Phase 1b study of combined selinexor and eribulin for the treatment of advanced solid tumors and triple-negative breast cancer. Cancer. 2023 Jul 15;129(14):2201-2213.	60	A phase 1b study in a single US institution of Selinexor (an oral XPO1 inhibitor that reduces nuclear-cytoplasmic trafficking of cancer-related proteins) combined with other standard treatments including eribulin. The majority of the 31 patients had a breast tumour (65%). Vaginal carcinoma and sarcoma patients were also included. The primary objective was the dose-limiting toxicity and maximum tolerated dose. 11 patients had serious adverse events. The ORR was 10%.	Median OS 12 months<br>(95% CI's not published).
Liu B, Liu L, Ran J, et al. A randomized trial of eribulin monotherapy versus eribulin plus alotinib in patients with locally recurrent or metastatic breast cancer. ESMO Open. 2023 Jun;8(3):101563.	Not published	A phase II study in 80 patients in a single Chinese centre of eribulin either alone or in combination with alotinib (10 or 12 mg for 14 days) for HER2-negative metastatic breast cancer. The primary endpoint was PFS. Overall response rate in those treated with eribulin and alotinib was 54.4%. The median PFS was 5.1 months (95% CI 4.3-6.9 months) for combination treatment and 3.5 months (95% CI 2.8-5.5 months) for eribulin alone (p=0.038). The addition of alotinib to eribulin did not increase the incidence of all grades of adverse events.	OS not reached. Median PFS 3.5 months (95% CI 2.8-5.5).
Hasler-Strub U, Mueller A, Li Q, Thuerlimann B, et al. Eribulin as first-line treatment in older patients with advanced breast cancer: A multicenter phase II trial [SAKK 25/14]. J Geriatr Oncol. 2023 Jan;14(1):101372.	76	This single-arm two-stage phase II trial aimed to study reduced dosing regimens in older patients (>70 years) with HER2-negative ER-positive metastatic breast cancer being treated in the first line. Eribulin was started at 1.1 mg/m^2 on days 1 and 8 of a 21-day cycle and dose-reduced according to toxicity. 77 patients enrolled across 18 Swiss hospitals. Median follow-up was 25.6 months.<br>The median PFS was 5.4 months. Two patients had a complete response. 62% of patients experienced at least one grade 3 toxicity. The authors stated that the efficacy of eribulin at lower doses was not equal to that of standard doses.	Median OS 16.1 months (95% CI 13.5-26.9).
La Verde N, Damia G, Garrone O, et al. Tolerability of Eribulin and correlation between polymorphisms and neuropathy in an unselected population of	60	A single-arm phase IV study of 180 patients with metastatic breast cancer (any receptor status) across 20 Italian centres who received eribulin (1.23 mg/m^2 on days 1 and 8 of a 21-day cycle) between 2014 and 2018. The median follow-up was 15.4 months. Tolerability was the primary outcome including quality of life assessments.	Median OS 12 months.<br>OS 79.1% and 49.8% at 6 and 12 months respectively.

Meta-analyses, randomised controlled trials and early phase trials of eribulin (56)			
**Publication** (Newest first)	**Median age (years)**	**Summary of findings**	**Reported survival outcomes** for eribulin-treated patients (unless otherwise stated)
female patients with metastatic breast cancer: results of the multicenter, single arm, phase IV PAINTER study. Breast Cancer Res. 2022 Oct 28;24(1):71.		42.4% of patients experienced at least one severe toxicity. There was an association of previous neuropathy with experiencing higher grade neuropathy on eribulin (p=0.0002). There was no difference in global health status between baseline and 3rd cycle questionnaires.	
Masuda N, Ono M, Mukohara T, et al. Phase 1 study of the liposomal formulation of eribulin (E7389-LF): Results from the breast cancer expansion cohort. Eur J Cancer. 2022 Jun;168:108-118.	59	28 Japanese patients with metastatic HER2-negative breast cancer received liposomal eribulin (2.0 mg/m^2 once every 3 weeks) as part of a previous phase I study. This was a follow-up study of efficacy and outcomes. The overall response rate was 35.7% (95% CI 18.6-55.9). 92.9% of patients experienced adverse events. The median PFS was 5.7 months (95% CI 11.5-43.1).	Median OS 18.3 months (95% CI 13.2-undefined).
Liu J, Wang Y, Tian Z. Multicenter phase II trial of Camrelizumab combined with Apatinib and Eribulin in heavily pretreated patients with advanced triple-negative breast cancer. Nat Commun. 2022 May 31;13(1):3011.	47	In this phase II trial 46 patients from a number of centres in China received camrelizumab, apatinib and eribulin for heavily pre-treated metastatic TNBC. ORR was the primary outcome measure. The ORR was 37.0% (95% CI 23.2-52.5). The median PFS was 8.1 (95%CI 4.6-10.3) months. Heavily pre-treated or PD-L1 negative patients received response benefit from multi-agent regimens.	OS not reported. Median PFS 8.1 months (95% CI 4.6-10.3).
Takashima T, Nishimura S, Kawajiri H, et al. An Exploratory Phase II Study of Eribulin Re-challenge After Short Term Therapy of 5-Fluorouracil for HER2 Negative, Advanced or Recurrent Breast Cancer. Anticancer Res. 2021 Oct;41(10):5007-5014.	64	15 treatment-naïve patients with metastatic HER2-ve breast cancer received eribulin in the first-line setting across 8 centres in Japan. Eribulin was received for 18 weeks and switched to capecitabine, in the absence of progression, for 4 weeks. Eribulin was then re-challenged. 12 patients started the re-challenge phase. 3 out of these 12 had progressive disease following this. Haematological adverse events were seen in all patients. The study failed to recruit and report it's primary outcome measures.	Median OS not reported. Median PFS 13 weeks (in the 12 patients who continued to eribulin re-challenge).
Cho EH, Kim JY, Im SA, et al. Potential role of CMPK1, SLC29A1, and TLE4 polymorphisms in gemcitabine-based chemotherapy in HER2-negative metastatic breast cancer patients: pharmacogenetic study results from the prospective randomized phase II study of eribulin plus gemcitabine versus paclitaxel plus gemcitabine (KCSG-BR-13-11). ESMO Open. 2021 Oct;6(5):100236.	50	In conjunction with a phase II trial of eribulin plus gemcitabine versus paclitaxel plus gemcitabine, this separate analysis looked at genetic polymorphisms and gemcitabine metabolism and outcomes in 45 patients with metastatic HER2-negative breast cancer. The primary outcome measure was the association of PFS with genetic polymorphisms thought to be associated with gemcitabine metabolism. No survival data were reported for eribulin plus gemcitabine patients alone.	OS across all polymorphisms ranged between 15.4 and 18.0 months. Survival data not reported for eribulin-only patients.

Meta-analyses, randomised controlled trials and early phase trials of eribulin (56)			
**Publication** (Newest first)	**Median age (years)**	**Summary of findings**	**Reported survival outcomes** for eribulin-treated patients (unless otherwise stated)
Keenan TE, Guerriero JL, Barroso-Sousa R, Li T, O'Meara T, Giobbie-Hurder A, Tayob N, Hu J, Severgnini M, et al. Molecular correlates of response to eribulin and pembrolizumab in hormone receptor-positive metastatic breast cancer. Nat Commun. 2021 Sep 21;12(1):5563.	57	This was a sub study looking at the molecular analysis of patients included in a randomised phase II trial of eribulin and pembrolizumab versus eribulin alone. 88 patients with metastatic ER-positive breast cancer were enrolled. The median follow-up was 25.8 months.<br>The primary aim of this study was identifying molecular mechanisms contributing to immune checkpoint inhibitor resistance in hormone receptor positive disease. Authors found lower levels of immune infiltration and greater tumour heterogeneity and oestrogen signalling in resistant tumours.	Median OS 14.3 months (95% CI 10.4-19.0; pembrolizumab + eribulin) versus 13.1 months (95% CI 9.4-19.4; eribulin alone) (HR 0.95, 95%CI 0.59-1.55, p=0.84)
Balch SM, Vaz-Luis I, Li T, etal. A phase II study of efficacy, toxicity, and the potential impact of genomic alterations on response to eribulin mesylate in combination with trastuzumab and pertuzumab in women with human epidermal growth factor receptor 2 (HER2)+ metastatic breast cancer. Breast Cancer Res Treat. 2021 Sep;189(2):411-423.	53	A single-centre phase II study of eribulin and trastuzumab-pertuzumab in 24 pre-treated patients with HER2-positive metastatic breast cancer. The primary outcome measure was ORR. The trial was stopped early due to slow accrual. The ORR was 26.3% (95% CI 9.2-51.2%). Median follow-up was 62 months. The most common adverse event was fatigue seen in 52.6% of patients.	Median OS 28.4 months and 19.9 months (95% CI 11.2-65.0) in patients with and without prior treatment with trastuzumab-pertuzumab respectively.
Tanni KA, Truong CB, Johnson BS, et al. Comparative effectiveness and safety of eribulin in advanced or metastatic breast cancer: a systematic review and meta-analysis. Crit Rev Oncol Hematol. 2021 Jul;163:103375.	Not published	This systematic review and meta-analysis of studies of eribulin versus control chemotherapy in locally advanced or metastatic breast cancer aimed to evaluate the clinical benefit and harms of eribulin.<br>13 studies were included in the review. The risk of neutropenia with eribulin versus non-eribulin regimens was statistically significantly higher (RR 1.68 (95% CI 1.04-2.73)).	OS HR 0.77 (95% CI 0.67-0.88) for eribulin versus non-eribulin regimens.
Aogi K, Watanabe K, Kitada M, et al. Clinical usefulness of eribulin as first- or second-line chemotherapy for recurrent HER2-negative breast cancer: a randomized phase II study (JBCRG-19). Int J Clin Oncol. 2021 Jul;26(7):1229-1236.	58	A randomised phase II trial of eribulin versus physicians choice as first- or second-line chemotherapy for recurrent HER2-negative breast cancer in 21 Japanese hospitals. 58 patients were recruited between 2013 and 2017. The primary endpoint was PFS.<br>The median follow-up was 14.0 months. The most common adverse event was neutropenia.	OS not reported. Median PFS 6.6 months (95% CI 5.0-8.1) versus 4.2 months (95% CI 0.8-7.6) in eribulin-treated versus physician choice-treated patients respectively (HR 0.72 95% CI 0.40-1.30, p=0.276).
Chalasani P, Farr K, Wu V, et al. Single arm, phase two study of low-dose metronomic eribulin in metastatic breast cancer.	59	In this single arm phase II trial of low dose eribulin in metastatic breast cancer, 50 patients received eribulin. Clinical benefit was reported in 49% of patients. The median PFS was 3.5 months (95% CI 2.6-4.8).	Median OS 14.3 months (95% CI 12.2-18.7).

Meta-analyses, randomised controlled trials and early phase trials of eribulin (56)			
**Publication** (Newest first)	**Median age (years)**	**Summary of findings**	**Reported survival outcomes** for eribulin-treated patients (unless otherwise stated)
Breast Cancer Res Treat. 2021 Jul;188(1):91-99.			
Zhao Q, Hughes R, Neupane B, et al. Network meta-analysis of eribulin versus other chemotherapies used as second- or nlater-line treatment in locally advanced or metastatic breast cancer. BMC Cancer. 2021 Jun 30;21(1):758.	50-60	This network meta-analysis of 7 randomised trials of eribulin versus other chemotherapies used as second- or later- line treatment in locally advanced/metastatic breast cancer included 4494 patients. Results showed that eribulin may provide an overall survival benefit in the locally advanced/metastatic breast cancer populations compared to standard treatments.	OS HR 0.81 (95% CI 0.66–0.99) for eribulin versus physicians choice. Eribulin versus gemcitabine and vinorelbine (HR 0.62; 95% CI 0.42–0.90).
Tolaney SM, Kalinsky K, Kaklamani VG, et al. Eribulin Plus Pembrolizumab in Patients with Metastatic Triple-Negative Breast Cancer (ENHANCE 1): A Phase Ib/II Study. Clin Cancer Res. 2021 Jun 1;27(11):3061-3068.	56	This open-label, single arm, phase Ib/II study in the United States compared eribulin plus pembrolizumab in patients with metastatic triple-negative breast cancer. This study included 167 patients. Patients with PD-L1 positive tumours had numerically higher objective response rate than those with PD-L1- negative tumours. They concluded eribulin plus pembrolizumab is generally well tolerated and shows promising anti-tumour activity in metastatic triple negative breast cancer.	Median OS 16.1 months (95% CI 13.3-18.5) Median OS 17.4 months (95% CI 13.2-21.0) in patients with no prior treatment. Median OS 15.5 months (95% CI 12.5-18.7) in patients with 1-2 lines of prior treatment.
Pérez-García JM, Llombart-Cussac A, G Cortés M, et al. Pembrolizumab plus eribulin in hormone-receptor-positive, HER2-negative, locally recurrent or metastatic breast cancer (KELLY): An open-label, multicentre, single-arm, phase II trial. Eur J Cancer. 2021 May;148:382-394.	53	This was a single-arm, multi-centre, phase II trial of eribulin plus pembrolizumab in 44 women with hormone-receptor positive locally recurrent or metastatic breast cancer in Spain. The primary objective was to assess clinical benefit. Eribulin plus pembrolizumab demonstrated encouraging anti-tumour activity with clinical benefit achieved in 25 women (56·8%; 95% CI 41·0–71·7).	1-year OS 59·1% (95% CI 45·8–76·2). Median PFS 6 months (95% CI 3·7–8·4).
De Angelis C, Bruzzese D, Bernardo A, et al. Eribulin in combination with bevacizumab as second-line treatment for HER2-negative metastatic breast cancer progressing after first-line therapy with paclitaxel and bevacizumab: a multicenter, phase II, single arm trial (GIM11-	56	This multicentre, single-arm, phase II study of eribulin plus bevacizumab in HER2-negative metastatic breast cancer progressing after first line treatment, evaluated 58 women for efficacy. The clinical benefit rate was 32.8% (95% CI 21.3-46.0). The results suggest that second line treatment with bevacizumab in combination with eribulin has meaningful clinical activity.	Median OS 14.8 months (95% CI 12.6-22.8). Median PFS 6.2 months (95% CI 4.0-7.8).

Meta-analyses, randomised controlled trials and early phase trials of eribulin (56)			
**Publication** (Newest first)	**Median age (years)**	**Summary of findings**	**Reported survival outcomes** for eribulin-treated patients (unless otherwise stated)
BERGI). ESMO Open. 2021 Apr;6(2):100054.			
Pellegrino B, Cavanna L, Boggiani D, et al. Phase II study of eribulin in combination with gemcitabine for the treatment of patients with locally advanced or metastatic triple negative breast cancer (ERIGE trial). Clinical and pharmacogenetic results on behalf of the Gruppo Oncologico Italiano di Ricerca Clinica (GOIRC). ESMO Open. 2021 Feb;6(1):100019.	56	A multicentre phase II study evaluating the combination of eribulin plus gemcitabine as first or second line treatment of locally advanced or metastatic triple negative breast cancer. The primary endpoint was ORR. 83 patients were enrolled, the ORR was 37.3% (31 patients) (90% CI 28.5-46.9). The clinical benefit rate was 48.8% (90% CI 39.2-58.4). This combination showed promising activity in metastatic TNBC patients.	Median OS 14.5 months (95% CI 10.1-19.8) Median PFS 5.1 months (95% CI 4.1-6.9).
Yamashita T, Kawaguchi H, Masuda N, et al. Efficacy of the eribulin, pertuzumab, and trastuzumab combination therapy for human epidermal growth factor receptor 2-positive advanced or metastatic breast cancer: a multicenter, single arm, phase II study (JBCRG-M03 study). Invest New Drugs. 2021 Feb;39(1):217-225	56	This was a multicentre, open-label, single arm phase II trial of eribulin, pertuzumab and trastuzumab for HER2 positive locally advanced or metastatic breast cancer. The primary endpoint was PFS. 50 patients were included in the study. In patients with HER2-positive MBC, eribulin, pertuzumab, and trastuzumab combination therapy exhibited substantial antitumor activity with an acceptable safety profile.	OS not reported. Median PFS 9.2 months (95% CI 7.0-11.4).
Filho OM, Giobbie-Hurder A, Lin NU, et al. A dynamic portrait of adverse events for breast cancer patients: results from a phase II clinical trial of eribulin in advanced HER2-negative breast cancer. Breast Cancer Res Treat. 2021 Jan;185(1):135-144.	56	A single arm, open-label phase II study of eribulin as first-or second-line for metastatic hormone receptor-positive/HER2-negative or TNBC. The primary endpoint was ORR. 83 patients were enrolled. The ORR in the hormone-receptor-positive/HER2-negative and TNBC cohorts was 35.6% (90% CI 24–39%) and 13.2% (90% CI 5–26%), respectively. Toxicity analysis revealed high levels of fatigue, alopecia, and neuropathy. Overall eribulin showed activity in HER2-negative breast cancer.	OS not reported. Median PFS 6.2 months (90% CI 5.9–8.7 months) in the hormone-receptor-positive/HER2-negative cohort and 4.0 months (90% CI 3.5–4.8 months) in the TNBC cohort.
Hardy-Bessard AC, Brocard F, Clatot F, et al. First-line bevacizumab and eribulin combination therapy for HER2-negative metastatic breast cancer: Efficacy and safety in the GINECO phase II ESMERALDA study.	59	A single-arm phase II study of first-line bevacizumab and eribulin for HER2-negative metastatic breast cancer. The primary endpoint was non-progression rate at 1-year. 61 patients were enrolled and received a median of six eribulin cycles. The non-progression rate at 1 year was 32% (95% CI 20-43%). ORR was 47% (95% CI 34-60%). First-line eribulin/bevacizumab showed activity with an acceptable safety profile.	OS not reported. Median PFS 8.3 months (95% CI 7.0-9.6 months).

Meta-analyses, randomised controlled trials and early phase trials of eribulin (56)			
**Publication**<br>(Newest first)	**Median age (years)**	**Summary of findings**	**Reported survival outcomes** for eribulin-treated patients (unless otherwise stated)
Breast. 2020 Dec;54:256-263.			
Tolaney SM, Barroso-Sousa R, Keenan T, et al. Effect of Eribulin With or Without Pembrolizumab on Progression-Free Survival for Patients With Hormone Receptor-Positive, ERBB2-Negative Metastatic Breast Cancer: A Randomized Clinical Trial. JAMA Oncol. 2020 Oct 1;6(10):1598-1605.	57	A multicentre phase II randomised trial comparing the efficacy of eribulin plus pembrolizumab to eribulin alone in patients with HR-positive, HER2-negative metastatic breast cancer. The primary endpoint was PFS. 88 patients started protocol therapy, and the median follow up was 10.5 months. Median PFS did not differ between the two arms (4.1 (95% CI 3.5-6.2) vs 4.2 months (95% CI 3.7-6.1)) (HR = 0.80; 95% CI 0.50-1.26; p=0.33), demonstrating no significant difference between the arms.	Median OS 13.4 months (95% CI 10.4-undefined; for eribulin and pembrolizumab treated patients)<br>Median OS 12.5 months (95% CI 8.6-undefined; for those receiving eribulin alone)<br>(HR = 0.87; 95% CI 0.48-1.59; p=0.65).
Miyoshi Y, Yoshimura Y, Saito K, et al.<br>High absolute lymphocyte counts are associated with longer overall survival in patients with metastatic breast cancer treated with eribulin-but not with treatment of physician's choice-in the EMBRACE study. Breast Cancer. 2020 Jul;27(4):706-715.	55	A sub-analysis of a phase III open-label study (EMBRACE) which compared eribulin to physician's choice (TPC) in 762 metastatic breast cancer patients. This study looked at the baseline absolute lymphocyte counts (ALCs) and neutrophil-to-lymphocyte ratio (NLR), which is a marker of immune status. Eribulin prolonged overall survival versus physician's choice in patients with baseline ALC ≥ 1500/μl (HR = 0.586; 95% CI 0.437–0.784; *P*<0.001).	Median OS 13.1 vs 10.6 months in the eribulin group versus the TPC group respectively (HR =0.81; 95% CI 0.66–<br>0.99; p=0.041).
Smith J 2nd, Irwin A, Jensen L, et al.<br>Phase II Study of Eribulin Mesylate<br>Administered Biweekly in Patients With Human Epidermal Growth Factor Receptor-2-negative Metastatic Breast Cancer. Clin Breast Cancer. 2020 Apr;20(2):160-167.	64	This open-label, single-arm, multicentre phase II study including 58 patients with previously treated HER2-negative metastatic breast cancer assessed a modified bi-weekly dosing schedule of eribulin for efficacy. The primary endpoints were ORR and disease control rate (DCR). The ORR was 12% (95% CI 5-24). The results were similar to those associated with the currently approved eribulin schedule reported in EMBRACE.	OS not reported.<br>Median PFS 3.6 months (95% CI 2.9-4.1 months).
Lee JS, Yost SE, Blanchard S, et al.<br>Phase I clinical trial of the combination of eribulin and everolimus in patients with metastatic triple-negative breast cancer. Breast Cancer Res. 2019 Nov 8;21(1):119.	55	A phase I clinical trial of the combination of eribulin and everolimus in patients with metastatic triple negative breast cancer. The primary objective was to evaluate the safety and toxicities of this combination. 27 patients were enrolled receiving a median of 4 cycles. Among 25 eligible patients, 9 patients (36%) achieved the best response as partial response, 9 (36%) had stable disease, and 7 (28%) had progression. The median time to progression was 2.6 months (95% CI 2.1-4.0).	Median OS 8.3 months (95% CI 5.5-undefined).

Meta-analyses, randomised controlled trials and early phase trials of eribulin (56)			
**Publication**<br>(Newest first)	**Median age (years)**	**Summary of findings**	**Reported survival outcomes** for eribulin-treated patients (unless otherwise stated)
Iwasa T, Tsurutani J, Watanabe S, et al. Multicentre, phase II study of eribulin in combination with S-1 in patients with advanced breast cancer. BMC Cancer. 2019 Oct 16;19(1):962.	54	This multicentre, phase II study of eribulin in combination with S-1 enrolled 33 patients with advanced breast cancer. The primary endpoint was ORR. Confirmed ORR was 33.3% (95% CI 17.3-52.80). This study showed this combination was safe and effective for poor prognosis advanced breast cancer patients.	Median OS not reached.<br>Median PFS 7.5 months (95% CI 4.0-14.3).
Di Cosimo S, La Verde N, Moretti A, et al. Neoadjuvant eribulin mesylate following anthracycline and taxane in triple negative breast cancer: Results from the HOPE study. PLoS One. 2019 Aug 7;14(8):e0220644.	43	This multicentre, prospective, non-randomized, open-label, single-arm, two stage, phase II trial evaluated the antitumor activity of eribulin following anthracycline and taxane (AT). 13 patients were enrolled with the primary objective to assess pathological complete response rate of eribulin combined with an AT-based regimen. AT followed by eribulin induced clinical complete and partial responses in 11/13 patients (85%) and a pathological complete response in 3/13 (23%). Median follow-up was 49 months.	OS not reported.<br>Event free survival (EFS) 61%.
Inoue K, Ninomiya J, Saito T, et al.<br>Eribulin, trastuzumab, and pertuzumab as first-line therapy for patients with HER2-positive metastatic breast cancer: a phase II, multicenter, collaborative, open-label, single-arm clinical trial. Invest New Drugs. 2019 Jun;37(3):538-547.	57	A phase II, multi-centre, open-label, single-arm clinical trial examining the efficacy and safety of triple therapy with eribulin, trastuzumab and pertuzumab in 25 women with HER2-positive metastatic breast cancer. The primary endpoint was ORR and the secondary endpoints included TTF and PFS. The median TTF with eribulin was 9.1 months (95% CI 4.3–13.9). The ORR was 80.0% (95% CI 59.3–93.2).	OS not reported.<br>Median PFS 23.1 months (95% CI 14.4–31.8).
Yuan P, Hu X, Sun T, et al. Eribulin mesilate versus vinorelbine in women with locally recurrent or metastatic breast cancer: A randomised clinical trial. Eur J Cancer. 2019 May;112:57-65.	50	This phase III open-label, randomised, parallel-group, multicentre clinical trial to evaluate the efficacy and safety of eribulin monotherapy, relative to vinorelbine, in Chinese women with locally recurrent/metastatic breast cancer, enrolled 530 women. Median PFS was 2.8 months in both treatment arms. The ORR was 30.7% (95% CI 25.2%-36.6%) with eribulin and 16.9% (95% CI 12.6%-22.0%) with vinorelbine ($p<0.001$).	Median OS 13.4 months (95% CI 11.5–16.2) with eribulin and 12.5 months (95% CI 10.6–16.6) with vinorelbine (HR 1.03, 95% CI 0.80-1.31, $p=0.838$).
Bischoff J, Barinoff J, Mundhenke C, et al.<br>A randomized phase II study to determine the efficacy and tolerability of two doses of eribulin plus lapatinib in trastuzumab-pretreated patients with HER-2-positive metastatic breast cancer (E-VITA). Anticancer Drugs. 2019 Apr;30(4):394-401.	54	A randomised phase II study to determine the efficacy and tolerability of two doses of eribulin plus lapatinib in 43 trastuzumab-pre-treated patients with HER2-positive metastatic breast cancer. Time to progression and tolerability were defined as primary end points. At a median follow-up of 28.7 months, the median time to progression was 8.1 months (95% CI 4.8-9.4) in those receiving eribulin 1.23 mg/m^2 on days 1 and 8 and 6.5 months (95% CI 4.6–13.4) with a 3-weekly schedule.	OS similar in both dosing groups: 23.1 months (95% CI 12.5–35.0) and 23.2 months (95% CI 13.7–30.1) respectively.

Meta-analyses, randomised controlled trials and early phase trials of eribulin (56)			
**Publication** (Newest first)	**Median age (years)**	**Summary of findings**	**Reported survival outcomes** for eribulin-treated patients (unless otherwise stated)
Ortega V, Antón A, Garau I, et al. Phase II, Multicenter, Single-arm Trial of Eribulin as First-line Therapy for Patients With Aggressive Taxane-pretreated HER2-Negative Metastatic Breast Cancer: The MERIBEL Study. Clin Breast Cancer. 2019 Apr;19(2):105-112.	47	A phase II multicentre, single-arm trial of eribulin as first line for taxane pre-treated HER2-negative metastatic breast cancer. The primary endpoint was time to progression. The median follow-up was 12.7 months. The median investigator-assessed time to progression was 4.1 months (95% CI 3.2-6.2).	Median OS not reached.<br>1-year OS 68.3% (95% CI 56.5-82.5).
Manso L, Moreno Antón F, Izarzugaza Perón Y, et al. Safety of eribulin as third-line chemotherapy in HER2-negative, advanced breast cancer pre-treated with taxanes and anthracycline: OnSITE study. Breast J. 2019 Mar;25(2):219-225.	58	A multicentre phase II study of anthracyclines and taxanes in patients with pre-treated HER2-negative advanced breast cancer, planned to receive eribulin as third-line chemotherapy. Efficacy, in terms of OS, PFS and the dynamics of circulating tumor cells (CTCs) during treatment were assessed. 59 patients were enrolled with a median PFS of 4 months (95% CI 3.1-5.9). Median OS was significantly longer in <5 baseline CTC patients compared to ≥5 baseline CTC patients (13.1 months (95% CI 11.8-not reached) vs 12.5 months (95% CI 7.6-not reached); p= 0.045).	Median OS 13.6 months (95% CI 11.8-not reached).
Yonemori K, Shimomura A, Yasojima H, et al. A phase I/II trial of Olaparib tablet in combination with eribulin in Japanese patients with advanced or metastatic triple-negative breast cancer previously treated with anthracyclines and taxanes. Eur J Cancer. 2019 Mar;109:84-91.	52 (Phase I), 46 (Phase II)	A multicentre phase I/II trial of olaparib plus eribulin in Japanese patients with advanced or metastatic TNBC to determine the recommended phase II dose and to examine efficacy and safety. The recommended dose was established as 300mg twice daily for Olaparib and 1.4mg/m^2 for eribulin. 24 patients were enrolled in each phase, with a median PFS of 4.2 months (95% CI 3.0-7.4).	Median OS 14.5 months (95% CI 4.8-22.0).
Sakaguchi K, Nakatsukasa K, Koyama H, et al. Phase II Clinical Trial of First-line Eribulin Plus Trastuzumab for Advanced or Recurrent HER2-positive Breast Cancer. Anticancer Res. 2018 Jul;38(7):4073-4081.	63	A phase II single-arm study to assess the efficacy and safety of a first-line regimen of eribulin plus trastuzumab for untreated advanced HER2-positive breast cancer patients. The primary endpoint was the response rate (RR). 28 patients were enrolled with a response rate of 53.6% (95% CI 36.6-69.9) and a median duration of response of 344 days (95% CI 237-680).	Median OS not reached.
Ohtani S, Nakayama T, Yoshinami T, et al. Bi-weekly eribulin therapy for metastatic breast cancer: a multicenter phase II prospective study (JUST-STUDY). Breast Cancer. 2018 Jul;25(4):438-446.	60	A multicentre phase II prospective study to investigate whether bi-weekly eribulin is safe and effective in metastatic breast cancer patients. 88 patients enrolled; 42 patients received bi-weekly eribulin with 40 receiving standard therapy. The ORR in the whole population was 19.3% (95% CI 11.7–29.1). The median TTF was 77 days (95% CI 70–95) in the overall population, 75 days (95% CI 69–119) in the standard group and 81.5 days (95% CI 73–107) in the bi-weekly group.	Median OS 427 days (95% CI 300–701).

Meta-analyses, randomised controlled trials and early phase trials of eribulin (56)			
**Publication** (Newest first)	**Median age (years)**	**Summary of findings**	**Reported survival outcomes** for eribulin-treated patients (unless otherwise stated)
Hayashida T, Jinno H, Mori K, et al. Phase II trial of eribulin mesylate as a first- or second-line treatment for locally advanced or metastatic breast cancer: a multicenter, single-arm trial. BMC Cancer. 2018 Jun 28;18(1):701.	66	This Japanese open-label, multicentre phase II study was designed to evaluate the efficacy and safety of eribulin as a first- or second-line treatment for patients with metastatic breast cancer. The primary objective was to determine the overall response rate. 35 patients were enrolled and the ORR was 43.8% (95% CI 26.5-61.0).	OS not reported. Median PFS 8.3 months (95% CI 7.1-9.4).
Inoue K, Ninomiya J, Saito T, et al. Induction therapy with paclitaxel and bevacizumab followed by switch maintenance therapy with eribulin in Japanese patients with HER2-negative metastatic breast cancer: a multicenter, collaborative, open-label, phase II clinical study for the SBCCSG 35 investigators. BMC Cancer. 2018 Jun 20;18(1):671.	66	A multicentre, collaborative, open-label, phase II clinical study of induction therapy with paclitaxel and bevacizumab followed by a switch to maintenance therapy with eribulin in Japanese patients with HER2-negative metastatic breast cancer. The primary endpoint was TTF, 51 patients were enrolled. Median TTF was 9.2 months (95% CI 7.3–11.1), median PFS was 10.7 months (95% CI 9.6–11.8).	Median OS 20 months (95% CI 16.0–24.0).
Pernas S, Martin M, Kaufman PA, et.al Balixafortide plus eribulin in HER2-negative metastatic breast cancer: a phase 1, single-arm, dose-escalation trial. Lancet Oncol. 2018 Jun;19(6):812-824.	56	A phase I single-arm dose-escalation trial of balixafordtide plus eribulin in 56 HER2-negative metastatic breast cancer of in Spain and the USA. The highest dose was established as eribulin 1·4 mg/m^2 on days 2 and 9, and balixafortide 5·5 mg/kg on days 1-3 and 8-10 of a 21-day cycle.	Median OS not reached. Median PFS 4·6 months (95% CI 3·1–5·7).
Kimura K, Iwamoto M, Tanaka S, et al. A phase II, multicenter, single-arm trial of eribulin as first- or second-line chemotherapy for HER2-negative advanced or metastatic breast cancer: evaluation of efficacy, safety, and patient-reported outcomes. Cancer Chemother Pharmacol. 2018 May;81(5):923-933.	64	This phase II, open-label, single-arm, multicentre study in Japan investigated the efficacy and safety of eribulin for first- or second-line chemotherapy for HER2-negative MBC. Primary endpoint was overall response rate. 35 patients were enrolled and the ORR was 37.1% (95% CI 21.1-53.2%). The median PFS was 6.2 months (95% CI 2.7-9.4 months).	Median OS 21.4 months (95% CI 11.5-32.9).

Meta-analyses, randomised controlled trials and early phase trials of eribulin (56)			
**Publication** (Newest first)	**Median age (years)**	**Summary of findings**	**Reported survival outcomes** for eribulin-treated patients (unless otherwise stated)
Pivot X, Im SA, Guo M, Marmé F. Subgroup analysis of patients with HER2-negative metastatic breast cancer in the second-line setting from a phase 3, open-label, randomized study of eribulin mesilate versus capecitabine. Breast Cancer. 2018 May;25(3):370-374.	40-65	This subgroup analysis of patients with HER2-negative metastatic breast cancer in a phase III randomised trial comparing eribulin versus capecitabine included 392 patients and assessed progression free survival and overall survival outcomes. Median PFS was similar between arms, 4.2 (eribulin) vs 4.0 (capecitabine) months (HR: 0.86; 95% CI 0.69–1.08; p=0.192).	Median OS 16.1 vs 13.5 months in patients receiving eribulin versus capecitabine, respectively (HR 0.77, p=0.026).
Hattori M, Ishiguro H, Masuda N, et al. Phase I dose-finding study of eribulin and capecitabine for metastatic breast cancer: JBCRG-18 cape study. Breast Cancer. 2018 Jan;25(1):108-117.	47	This phase I dose-finding study of eribulin and capecitabine for metastatic breast cancer enrolled nine patients. A dosing schedule of eribulin at 1.4 mg/m^2 on days 1 and 8 combined with capecitabine 1000 mg/m^2 twice daily in a 2-weeks-on and 1-week-off schedule in a 21-day cycle was tolerable and chosen for further investigation in the phase II study.	OS not reported. Mean PFS 9.2 months (95% CI 1.07–17.33).
Park YH, Im SA, Kim SB, et al. Phase II, multicentre, randomised trial of eribulin plus gemcitabine versus paclitaxel plus gemcitabine as first-line chemotherapy in patients with HER2-negative metastatic breast cancer. Eur J Cancer. 2017 Nov;86:385-393.	50	This prospective randomised phase II, open-label, two-arm, multicentre study compared eribulin plus gemcitabine (EG) with paclitaxel plus gemcitabine (PG) chemotherapy as a first-line treatment in 118 patients with HER2-negative MBC. The PFS rate at 6 months, which was the primary end-point of this study, between the two arms was 72% and 73%, respectively (HR= 0.84; 95% CI 0.53-1.33; p=0.457).	Median OS not reached in the PG group, 21.2 months in the eribulin group (HR= 0.57; 95% CI 0.23-1.45; p=0.240).
Araki K, Fukada I, Yanagi H et al. First report of eribulin in combination with pertuzumab and trastuzumab for advanced HER2-positive breast cancer. Breast. 2017 Oct;35:78-84.	58	This single-arm, phase II trial investigated eribulin in combination with pertuzumab and trastuzumab for both taxane- and trastuzumab-pretreated HER2-positive advanced breast cancer patients. The pharmacokinetics of this combination were assessed and the primary endpoint was objective response rate. Pharmacokinetics of eribulin in this combination were similar to previous reports of eribulin monotherapy. ORR was 34.8% (95% CI 16.4-57.3).	OS not reported. Median PFS 42.6 weeks (95% CI 20.3-51.9).
Maeda S, Saimura M, Minami S, et al. Efficacy and safety of eribulin as first- to third-line treatment in patients with advanced or metastatic breast cancer previously treated with anthracyclines and taxanes. Breast. 2017 Apr;32:66-72.	54	In this phase II, open-label, single-arm study conducted at 14 sites in Japan, 47 women with HER2-negative advanced breast cancer received eribulin. The objective response rate was 17.0% (95% confidence interval, 7.6-30.8) and median PFS was 4.9 months (3.5-7.0). Subgroup analysis indicated that first-line treatment led to higher ORR and prolonged PFS and OS than second-/third-line treatment.	Median OS 17.4 months (10.1-not evaluable).

Meta-analyses, randomised controlled trials and early phase trials of eribulin (56)			
**Publication** (Newest first)	**Median age (years)**	**Summary of findings**	**Reported survival outcomes** for eribulin-treated patients (unless otherwise stated)
Park YH, Kim TY, Im YH, et al. Feasibility and Efficacy of Eribulin Mesilate in Korean Patients with Metastatic Breast Cancer: Korean Multi-center Phase IV Clinical Study Results. Cancer Res Treat. 2017 Apr;49(2):423-429.	51	This multicentre single-arm phase IV clinical trial assessed the efficacy and safety of eribulin in Korean patients with metastatic breast cancer. 101 patients were included with a median of three treatment cycles. Disease control rate was 51.3% (95% CI 39.6-63.0) and ORR was 17.1% (n=13).	OS not reported. Median PFS 2.36 months (95% CI 2.10-4.32).
Yardley DA, Reeves J, Dees EC, et al. Ramucirumab With Eribulin Versus Eribulin in Locally Recurrent or Metastatic Breast Cancer Previously Treated With Anthracycline and Taxane Therapy: A Multicenter, Randomized, Phase II Study. Clin Breast Cancer. 2016 Dec;16(6):471-479.e1.	57 (eribulin); 56 (ramicurimab +eribulin)	In this phase II study in the US 141 women with locally recurrent or metastatic breast cancer and previous anthracycline and taxane treatment were randomised to ramucirumab with eribulin or eribulin alone. Median PFS for ramucirumab with eribulin was 4.4 months (95% CI 3.1-6.7) compared with 4.1 months (95% CI 3.2-5.6) for eribulin (HR =0.83; 95% CI 0.56-1.23; p=0.35).	Median OS 13.5 months (95% CI, 10.4-17.9) versus 11.5 months (95% CI, 9.0-17.3) in patients who received ramucirumab with eribulin versus eribulin alone, respectively (HR 0.91; 95% CI 0.59-1.41; p=0.68).
Inoue K, Saito T, Okubo K, et al. Phase II clinical study of eribulin monotherapy in Japanese patients with metastatic breast cancer who had well-defined taxane resistance. Breast Cancer Res Treat. 2016 Jun;157(2):295-305.	55	This phase II Japanese multicentre single-arm study assessed the efficacy and safety of eribulin monotherapy in patients with MBC with taxane resistance. 51 patents received a median of 4 cycles. The clinical benefit rate was 39.2% and the rate of progressive disease was 49.0%. The median PFS was 3.6 months (95 % CI 2.6–4.6).	Median OS 11.7 months (95% CI 9.2–14.2).
Aftimos P, Polastro L, Ameye L, et al. Results of the Belgian expanded access program of eribulin in the treatment of metastatic breast cancer closely mirror those of the pivotal phase III trial. Eur J Cancer. 2016 Jun;60:117-24.	55	A multicentre single-arm study enabling expanded access to eribulin for patients with advanced breast cancer and no further treatment options. This study ran in Belgium, Canada and France. 154 patients were enrolled and received a median of 4 cycles. Median PFS was 3.2 months (95% CI 2.7–4.0). At the time of the analysis, 112 patients had died.	Median OS 11.3 months (95% CI 8.7–12.3).
Kaufman PA, Awada A, Twelves C, et al. Phase III open-label randomized study of eribulin mesylate versus capecitabine in patients with locally advanced or metastatic breast cancer previously treated with an anthracycline and a taxane. J Clin	54 (eribulin), 53 (capecitabine)	This phase III randomised trial of 1102 patients compared eribulin with capecitabine in patients with locally advanced or metastatic breast cancer who had received anthracycline and taxane based therapy. Median PFS times for eribulin and capecitabine were 4.1 and 4.2 months, respectively (HR 1.08; 95% CI 0.93-1.25; p=0.30). Eribulin was not shown to be superior to capecitabine with regard to OS or PFS.	Median OS 15.9 and 14.5 months, for eribulin and capecitabine respectively (HR 0.88; 95% CI 0.77-1.00; p=0.056).

Meta-analyses, randomised controlled trials and early phase trials of eribulin (56)			
**Publication** (Newest first)	**Median age (years)**	**Summary of findings**	**Reported survival outcomes** for eribulin-treated patients (unless otherwise stated)
Oncol. 2015 Feb 20;33(6):594-601.			

Abbreviations: PFS = progression free survival, OS = overall survival, ORR = objective/overall response rate, HER2 = human epidermal growth factor receptor 2, RR= risk ratio, CI = confidence interval, HR = hazard ratio, HR = hormone receptor, ER = oestrogen receptor, TNBC = triple negative breast cancer, MBC= metastatic breast cancer, PD-L1 = programmed cell death ligand one.

Table S7 - Observational, cohort and real-world studies of eribulin (19)

A search was performed on 11th September 2025 for studies published between 1st January 2015 until 11th September 2025 on patients with breast cancer where eribulin was studied in an observational, cohort or real-world setting. Studies were included if they were written in English, conducted in humans and included over 250 participants regardless of treatment arm. Studies without published survival data (overall survival, time to next treatment or death or progression free survival) were excluded as were trials that repeated outcomes reported elsewhere and included here.

Observational, cohort and real-world studies (19)			
**Publication** (Newest first)	**Median age (years)**	**Summary of findings**	**Reported survival outcomes** for eribulin-treated patients (unless otherwise stated)
Kimura T, Takami T, Piao Y et al. Treatment patterns and clinical outcomes in patients with hormone receptor-positive and human epidermal growth factor receptor 2-negative metastatic breast cancer treated with chemotherapy: a large-scale data analysis using the Japanese claims database. Breast Cancer Res Treat. 2025 May;211(1):233-244.	61	Of 2697 patients with metastatic HER2-negative, ER-positive breast cancer who were documented as receiving chemotherapy between 2017 and 2022 in Japanese medical claims data, 798 received eribulin with 36% (286 patients) of these receiving it as a first line drug. The primary outcome measure was time to next treatment or death. The median time to next treatment (TTNT) or death overall was 8.2 months for first line treatment and time to next treatment or death shortened with progressive lines of chemotherapy.	OS not reported. Median TTNT 7.3 months (eribulin as first line chemotherapy).

Observational, cohort and real-world studies (19)			
**Publication** (Newest first)	**Median age (years)**	**Summary of findings**	**Reported survival outcomes** for eribulin-treated patients (unless otherwise stated)
El Kaddissi A, Vernerey D, Falcoz A, et al. Prognostic Factors for Long-Term Eribulin Response in a Cohort of Patients With HER2-Negative Metastatic Breast Cancer. Clin Breast Cancer. 2024 Oct;24(7):e622-e632.e5.	61	The chemotherapy prescribing software of 7 French hospitals was retrospectively reviewed to identify women with HER2-negative metastatic breast cancer who received eribulin between 2011 and 2020. 374 patients were included in the analysis which identified low grade tumours, prior chemotherapy efficacy, absence of meningeal metastases and low ECOG status at the beginning of treatment as prognostic factors associated with eribulin prescription.	Median OS 8.5 months (95% CI 7.0-9.5)<br>Median PFS 3.2 months (95% CI 2.8-3.7).
Tolaney SM, Punie K, Carey LA et al. Real-world treatment patterns and outcomes in patients with HR+/HER2- metastatic breast cancer treated with chemotherapy in the United States. ESMO Open. 2024 Sep;9(9):103691.	61	1545 patients were identified from electronic health records in the United States who started first line chemotherapy for newly diagnosed HER2-negative, ER-positive metastatic breast cancer between 2011 and 2021. Overall survival was the primary outcome. Of 281 patients who received eribulin, 2% of these used eribulin first line, 16% used it fourth line. The authors found that survival decreases with each additional chemotherapy line.	Median OS 12.27 months (95% CI 6.55-19.67; eribulin as first line chemotherapy).
Kimura T, Takami T, Piao Y, et al. Treatment patterns and clinical outcomes in patients with metastatic triple-negative breast cancer: a large-scale data analysis using the Japanese claims database. Breast Cancer Res Treat. 2024 Jul;206(1):91-103.	66	A Japanese medical claims database was interrogated for women with newly diagnosed triple negative metastatic breast cancer between 2017 and 2022. 2236 patients were identified. 461 patients received eribulin. Eribulin was the most commonly used second- and third-line therapy (18.3% and 19.4% of the cohort respectively). The primary outcome measure was time to next treatment or death. Time to next treatment or death shortened as the therapy progressed.	OS not reported.<br>Median TTNT 8 months (95% CI 7.3-8.9; eribulin as first line chemotherapy).
Coe F, Misra V, McCabe Y, et al. Average duration of prior treatment lines predicts clinical benefit to eribulin chemotherapy in patients with metastatic breast cancer. Breast Cancer Res Treat. 2022 Feb;191(3):535-543.	56	Hospital records for 439 patients who received Eribulin for breast cancer at the Christie Hospital in Manchester UK between 2011 and 2018 were reviewed. Patients with any receptor status were accepted. Patients with longer duration on previous treatment lines had better outcomes on eribulin especially for those with TNBC.	Median OS 8.6 months (95% CI 7.4-9.8).
Jafri M, Kristeleit H, Misra V, et al. Eribulin Treatment for Patients with Metastatic Breast Cancer: The UK Experience - A Multicenter Retrospective Study. Oncology. 2022;100(12):666-673.	56	The hospital and chemotherapy records of 577 women with metastatic breast cancer and any receptor status who received at least one cycle of eribulin between 2011 and 2017 in one of 14 UK hospitals were reviewed. OS was the primary outcome. The median OS was higher among patients over 65 (>65 vs <65: 325 vs 285 days; p=0.028) and in patients who received eribulin after fewer prior lines of chemotherapy (≤2 vs. >2 prior: 328 days vs 264 days; p=0.042).	Median OS 288 days (95% CI 261-315).
Takahashi M, Inoue K, Mukai H, et al. Indices of peripheral leukocytes predict longer overall survival in breast cancer patients on eribulin in Japan. Breast Cancer. 2021 Jul;28(4):945-955	Not published	This was a post-marketing study of 1123 Japanese women with HER2-negative inoperable or recurrent breast cancer treated with eribulin. Patients were enrolled between 2014 to 2016 and followed up for 2 years. The main outcome measure was OS dependent on absolute lymphocyte count (ALC). The authors showed that patients with a baseline lymphocyte count of >1500/µL showed a prolonged OS compared to those with a lymphocyte count of <1500/µL indicating that lymphocyte count could be a predictor of response.	Median OS in patients with ALC >1500/µL 19.4 months versus 14.3 months in patients with ALC <1500/µL (HR 0.628; 95% CI 0.492-0.801).

Observational, cohort and real-world studies (19)			
**Publication** (Newest first)	**Median age (years)**	**Summary of findings**	**Reported survival outcomes** for eribulin-treated patients (unless otherwise stated)
Grinda T, Antoine A, Jacot W, et al. Evolution of overall survival and receipt of new therapies by subtype among 20 446 metastatic breast cancer patients in the 2008-2017 ESME cohort. ESMO Open. 2021 Jun;6(3):100114.	60	This was a retrospective cohort study of 20,446 women with information gathered from medical records across 18 French hospitals. The women commenced treatment for metastatic breast cancer of any receptor status between 2008 and 2016. The primary outcome was OS. Eribulin use did not exceed 32% in any year of the study. The authors found that OS has dramatically improved among HER2-negative patients over the study period.	Median OS 38.8 months (95% CI 38.1-39.7; patients treated with many chemotherapy regimens).
Lin YJ, Kuo CN, Ko Y. Effectiveness and healthcare costs of eribulin versus capecitabine among metastatic breast cancer patients in Taiwan. Breast. 2021 Jun;57:18-24.	54	A study of retrospective data from a Taiwanese routinely collected health data repository of 298 women with metastatic breast cancer (any receptor status) who had received 1 or more prior lines of chemotherapy. Patients enrolled between 2015 to 2016 and were followed up until 2017. The primary outcome was OS. Authors found that OS was equivalent to capecitabine when used as sixth- or later-line treatment.	Median OS 11.8 months (95% CI 11.5-13.5).
Mougalian SS, Kish JK, Zhang J, et al. Effectiveness of Eribulin in Metastatic Breast Cancer: 10 Years of Real-World Clinical Experience in the United States. Adv Ther. 2021 May;38(5):2213-2225	59	This was a chart-reviewed retrospective analysis of 513 US women with metastatic breast cancer treated with eribulin between 2011 and 2017 and followed up for 2 years with 2-or-more prior lines of chemotherapy. Primary outcome was ORR. The authors found that the outcomes for the real-world patients were comparable to those identified in clinical trials.	Median OS 10.6 months (95% CI 9.9-11.7).
Chabot I, Zhao Q, Su Y. Systematic review of Real-World effectiveness of eribulin for locally advanced or metastatic breast cancer. Curr Med Res Opin. 2020 Dec;36(12):2025-2036.	74	This was a systematic review of real-world studies on the effectiveness of eribulin. Studies were included if they were published between 2012 and 2019 and reported the use of eribulin in women with locally advanced and metastatic breast cancer (of any receptor status). Clinical trial populations were excluded. 34 trials met eligibility criteria. The authors found high variability in OS in included trials however they did find that, in real-world populations, median PFS and OS often exceeded that reported in clinical trials.	Median OS 6.9-28.0 months (range).
Park MH, Lee SJ, Noh WC et al. A nationwide, multicenter retrospective study on the effectiveness and safety of eribulin in Korean breast cancer patients (REMARK). Breast. 2020 Dec;54:121-126.	53	This was a retrospective analysis of 360 patients who received eribulin in 14 Korean hospitals for locally advanced or metastatic breast cancer (any receptor status) treated between 2014 and 2016. The primary outcome was PFS at 6 months. The authors found that eribulin was a safe and effective treatment for Korean populations.	Median OS 631 days (95% CI 571-761).
Kazmi S, Chatterjee D, Raju D, Hauser R, Kaufman PA. Overall survival analysis in patients with metastatic breast cancer and liver or lung metastases treated with eribulin, gemcitabine, or capecitabine. Breast Cancer Res Treat. 2020 Nov;184(2):559-565.	55	A retrospective analysis of women with stage 4 breast cancer (any receptor status) and lung or liver metastases treated with one of eribulin, gemcitabine or capecitabine as 3rd line therapy in 5 hospitals in the US between 2012 and 2018. OS was the primary outcome measure. 229 patients received eribulin. Median OS was highest in eribulin-treated patients when compared to gemcitabine and capecitabine-treated patients.	Median OS 9.8 months (95% CI 8.3-12.8).

Observational, cohort and real-world studies (19)			
**Publication** (Newest first)	**Median age (years)**	**Summary of findings**	**Reported survival outcomes** for eribulin-treated patients (unless otherwise stated)
Rugo HS, Dieras V, Cortes J et al. Real-world survival outcomes of heavily pretreated patients with refractory HR+, HER2-metastatic breast cancer receiving single-agent chemotherapy-a comparison with MONARCH 1. Breast Cancer Res Treat. 2020 Nov;184(1):161-172.	Not published	A study of retrospectively collected electronic health record data from 280 cancer clinics across the US that aimed to compare outcomes to the MONARCH-1 trial. 281 women were diagnosed with ER -positive, HER2-negative metastatic breast cancer between 2011 to 2018 and received either capecitabine, gemcitabine, eribulin or vinorelbine monotherapy as second or later line of chemotherapy. The primary outcome was OS. The authors suggest a survival advantage in heavily pre-treated patients treated with abemaciclib monotherapy compared to those treated with a single agent chemotherapy.	Median OS 13.6 months (95% CI 9.6-16.6; patients treated with capecitabine, gemcitabine, eribulin or vinorelbine).
Inoue K, Takahashi M, Mukai H, et al. Effectiveness and safety of eribulin in Japanese patients with HER2-negative, advanced breast cancer: a 2-year post-marketing observational study in a real-world setting. Invest New Drugs. 2020 Oct;38(5):1540-1549.	60	A prospective post-marketing observational study in 632 Japanese women with advanced HER2-negative breast cancer treated with eribulin between 2014 to 2016 and followed up for 2 years. The primary outcome measures were OS and time to treatment failure. The authors found that safety and efficacy of eribulin was similar to that reported in prior clinical and post-marketing studies.	Median OS 15.6 months (95% CI 13.8-17.6).
Pedersini R, di Mauro P, Amoroso V, et al. Efficacy of Eribulin mesylate in older patients with breast cancer: A pooled analysis of clinical trial and real-world data. J Geriatr Oncol. 2020 Jul;11(6):976-981.	72	A systematic review of studies up until 2019 in patients with metastatic breast cancer (of any receptor status) who received eribulin and who were >70 years. Only observational and retrospective studies were accepted. Efficacy outcomes (OS, PFS and RR) were the primary outcomes. 4 studies matched eligibility criteria including 232 patients. The authors commented that eribulin is effective and has a toxicity profile similar to that of selected populations in clinical trials.	Median OS 13.1 months (95% CI 9.2-16.9; pooled OS).
Jacot W, Heudel PE, Fraisse J, et al. Real-life activity of eribulin mesylate among metastatic breast cancer patients in the multicenter national observational ESME program. Int J Cancer. 2019 Dec 15;145(12):3359-3369.	Not published	A retrospective analysis of hospital records of 7412 patients treated for metastatic breast cancer (any receptor status) in 18 French hospitals between 2008 and 2014. Patients had received 2 lines of prior therapy. The primary end point was OS. Patients receiving eribulin in the second, third or fourth line presented a significantly better PFS and OS than patients receiving other chemotherapy agents.	Median OS 13.99 months (95% CI 11.47-17.48; for eribulin in the second-line setting).
Mougalian SS, Feinberg BA, Wang E et al. Observational study of clinical outcomes of eribulin mesylate in metastatic breast cancer after cyclin-dependent kinase 4/6 inhibitor therapy. Future Oncol. 2019 Dec;15(34):3935-3944.	65	214 patients with ER-positive HER2-negative metastatic breast cancer were treated with a CDK4/6 inhibitor between 2015 and 2017 and received eribulin thereafter were identified by individual practitioners in a number of western US institutions. Data was chart-reviewed by treating clinicians. Primary outcome measures were safety and PFS. The authors found that prior CDK 4/6 inhibitor use did not negatively impact eribulin activity and that safety was equal to published data on eribulin.	OS not reported. Median PFS 10.3 months (95% CI 7.8-17.5).

Observational, cohort and real-world studies (19)			
**Publication** (Newest first)	**Median age (years)**	**Summary of findings**	**Reported survival outcomes** for eribulin-treated patients (unless otherwise stated)
Watanabe J, Ito Y, Ohsumi S, et al. Safety and effectiveness of eribulin in Japanese patients with locally advanced or metastatic breast cancer: a post-marketing observational study. Invest New Drugs. 2017 Dec;35(6):791-799.	59	A post-marketing observational study in 325 Japanese centres of 951 patients with inoperable or recurrent breast cancer (any receptor status) receiving eribulin for the first time. Patients were enrolled between July 2011 to December 2011 and followed up for 1 year. The primary outcome measure was the frequency and intensity of adverse drug reactions. The authors found that safety and effectiveness profiles of eribulin were similar to those seen in clinical trials.	OS not reported. Median TTNT 127 days (95% CI 120-134).

Abbreviations: OS = overall survival, PFS = progression free survival, HER2 = human epidermal growth factor receptor 2, ER = oestrogen receptor, PFS = progression free survival, CDK = cyclin-dependent kinase, CI = confidence interval, TTNT = time to next treatment, HR = hazard ratio.
